# Associations of diabetes mellitus with/without diabetic retinopathy and cognitive outcomes in older adults: the potential role of the dietary inflammatory index in a multi-dataset observational study

**DOI:** 10.3389/fnut.2026.1775880

**Published:** 2026-05-12

**Authors:** Guiqian Huang, Sixuan Chen, Weixin He, Linyi Han, Ante Lou, Haiyue Zhu, Nan Liang, Lifei Sun, Yan Lin, Wenjun Wu, Yili Wu

**Affiliations:** 1 Wenzhou Key Laboratory of Basic and Translational Research for Mental Disorders, School of Mental Health, Key Laboratory of Alzheimer’s Disease of Zhejiang Province, Institute of Aging, Wenzhou Medical University, Wenzhou, Zhejiang, China; 2 Wenzhou Key Laboratory of Basic and Translational Research for Mental Disorders, School of Mental Health, Key Laboratory of Alzheimer’s Disease of Zhejiang Province, Institute of Aging, Oujiang Laboratory (Zhejiang Lab for Regenerative Medicine, Vision and Brain Health), Wenzhou Medical University, Wenzhou, Zhejiang, China; 3 Department of Psychiatry, The First Affiliated Hospital of Wenzhou Medical University, Wenzhou, Zhejiang, China; 4 First College of Clinical Medical, Wenzhou Medical University, Wenzhou, Zhejiang, China; 5 Department of Endocrinology, The First Affiliated Hospital of Wenzhou Medical University, Wenzhou, Zhejiang, China

**Keywords:** cognitive impairment, diabetes mellitus, diabetic retinopathy, dietary inflammatory index, older adults

## Abstract

**Background:**

Cognitive impairment is common among older adults with diabetes mellitus (DM). This study aimed to examine the associations between DM with/without diabetic retinopathy (DR) and adverse cognitive outcomes in older adults, and to assess the potential role of the dietary inflammatory profile in these associations.

**Methods:**

This observational study included older adults from the National Health and Nutrition Examination Survey (NHANES), UK Biobank (UKB), and the Wenzhou dataset. Cognitive outcomes were assessed using cognitive tests in NHANES and the Wenzhou dataset, and by all-cause dementia identified from ICD-10 codes in UKB, respectively. Dietary inflammatory index (DII) scores were derived from 24-h dietary recalls in NHANES, the Oxford WebQ in UKB, and the FFQ-25 in the Wenzhou dataset, respectively. Weighted multivariable-adjusted Poisson regression, robust Cox proportional hazards regression and conditional logistic regression were used in NHANES, UKB and the Wenzhou dataset, respectively. Exploratory mediation analyses were conducted in all three datasets to assess the potential role of DII in the associations between DM with or without DR and cognitive outcomes.

**Results:**

In NHANES, poorer cognitive performance was associated with DM and higher DII in the overall sample, while poorer cognitive performance was associated with DR and higher DII among participants with DM. Exploratory mediation analyses in NHANES suggested a potential role of DII in the associations between DM with/without DR and cognitive function impairment (CFI) (DM: 15.0% mediated; DR: 8.0% mediated). Restricted cubic spline analysis showed that higher DII levels were associated with higher odds of CFI. In UKB and the Wenzhou dataset, exploratory mediation analyses showed significant indirect effects of DII in the association between DM and dementia (4.1% mediated) and between DR and cognitive impairment (13.6% mediated), respectively.

**Conclusion:**

Dietary inflammatory profile reflected by DII may play a potential role in the associations of DM with/without DR and cognitive dysfunction. Further prospective studies and randomized controlled trials are needed to clarify causality and potential clinical benefit.

## 1 Introduction

According to current epidemiological analyses, about 589 million adults aged 20 to 79 around the world suffer from diabetes mellitus (DM) (1). In 2024, the estimated number of individuals with DM was 158.3 million (23.7%) among adults aged 65 years and older, and this number is projected to increase to 278 million by 2030 (1). Meanwhile, lifespans are increasing and approximately one-fifth of the U.S. population will be aged 65 years or older by 2030 (2). Over 20.5% of Americans aged 60 and older have been diagnosed with DM (3). The number of older adults with diagnosed diabetes in the United States rose from 4.7 million in 2000 to 11.0 million in 2018. Researchers project that this number will reach nearly 26.7 million by 2050 (4–6). Furthermore, DM is also a known risk factor for many microvascular and macrovascular complications in major organs. Its connection to cognitive impairment is also becoming more widely recognized (7–10). A recent review summarized evidence that diabetes is associated with neuropsychological problems, including cognitive decline and dementia (7). Therefore, attention should be paid to the emergence of cognitive impairment in older adults with DM to allow implementation of appropriate preventive measures.

Diabetic retinopathy (DR), a major complication of DM, affects approximately one-third of patients with DM and remains a leading cause of visual impairment worldwide (11). A diagnosis of DR is based on manifestations of vascular abnormalities in the retina, including increased vascular permeability, capillary occlusion, neovascularization, diabetic macular edema, and even loss of vision (12). DR is the most common and serious ocular complication (13). In addition, DR serves as a critical marker of the overall effectiveness of diabetes management, as it effectively reflects the progression and severity of DM (14, 15). Therefore, examining the association between DR and cognitive impairment may provide additional insight into how diabetes severity relates to cognitive health (16).

Diet is an important component of diabetes management, and dietary patterns among people with DM often differ from those of the general population because of the need for glycemic control and complication prevention. The relations between dietary patterns and cognitive ability are also complex and multidimensional. Different nutrients, such as carbohydrates, fats, proteins, and vitamins, are closely related to cognition (17, 18). The dietary inflammatory index (DII), developed in 2009 and updated in 2014, was designed to quantify the inflammatory potential of diet on the basis of its reported associations with six inflammatory biomarkers (19). Investigating the association of DII with cognitive outcomes in older adults with DM may help clarify whether the dietary inflammatory profile is relevant to diabetes-related cognitive vulnerability.

Although epidemiological studies suggest that DM is associated with adverse cognitive outcomes, the pathways underlying this association remain incompletely understood. In the present study, we focused on older adults and examined whether DII was associated with cognitive outcomes in individuals with DM and DR. Using NHANES as the primary dataset, with the UK Biobank and the Wenzhou dataset as complementary datasets, we aimed to evaluate whether similar association patterns could be observed across different populations and related cognitive outcomes.

## 2 Materials and methods

### 2.1 Study design and participants

Study participants were drawn from three observational datasets, the National Health and Nutrition Examination Survey (NHANES), the UK Biobank (UKB) and the Wenzhou dataset. In this study, NHANES was used as the primary dataset to examine the associations of DII with cognitive performance in older adults with DM and DR. The UKB and Wenzhou datasets were included as complementary datasets to assess whether similar association patterns could be observed across related cognitive outcomes.

The NHANES is a nationwide cross-sectional survey designed to assess the health and nutritional status of the civilian residents of the USA. Given that cognitive assessments were available only for participants aged 60 years and older during the year 1999–2002, 2011–2014, and 2017–2020 survey cycles, we included data for 3,632 participants aged ≥ 60 years between 2011 and 2014 from the NHANES database. To assess potential selection bias arising from complete-case analysis, we compared included and excluded participants using baseline characteristics available prior to exclusion. Each variable was summarized using all available observations, and denominators therefore varied across characteristics. The UKB is a population-based cohort that enrolled over 500,000 participants from 22 assessment centers across Scotland, England and Wales between 2006 and 2010 (20). To improve comparability in age range across cohorts, we included 217,401 participants aged ≥ 60 years at enrollment from the UKB. The Wenzhou dataset was derived from a hospital-based case–control study collected from the clinical database of patients with DM. This study enrolled DM patients aged ≥ 60 years who were admitted to the Department of Endocrinology at the First Affiliated Hospital of Wenzhou Medical University between October 2023 and October 2025. Ethical approval was obtained from the Ethics Committee of the First Affiliated Hospital of Wenzhou Medical University, and the studies were conducted in accordance with the Declaration of Helsinki. Informed consent was obtained from all participants.

**Figure 1:**
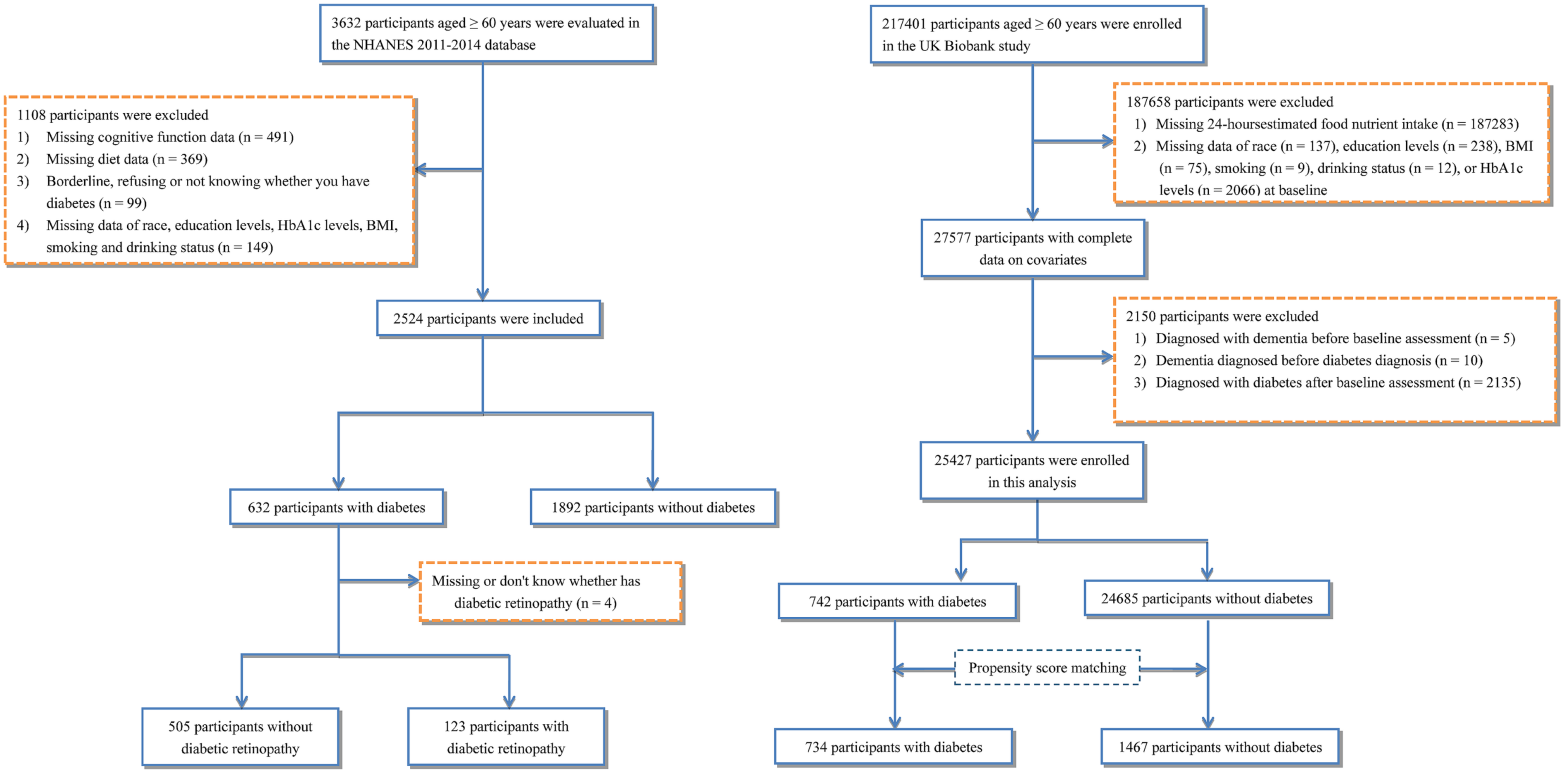
Flow diagram of the study and participants in NHANES and UK Biobank datasets. NHANES, National Health and Nutrition Examination Survey.

Demographic characteristics, lifestyle factors, and clinical covariates related to cognitive health were collected according to data availability in each dataset. A complete-case analytic approach was used after excluding participants with incomplete cognitive assessment, missing dietary data, and missing data on key covariates, including race, education level, HbA1c levels, body mass index (BMI), smoking status, and drinking status. Finally, a total of 2,524 participants from the NHANES dataset were included, comprising 632 participants with DM and 1,892 participants without DM (Figure 1). A total of 25,427 participants from the UKB dataset were included in the analysis, including 742 participants with DM and 24,685 participants without DM. Propensity score matching was performed to reduce baseline imbalance and potential confounding between the two groups, resulting in 734 participants with DM matched to 1,467 participants without DM (Figure 1). In the Wenzhou study, given the higher severity of DM and the increased incidence of cognitive impairment among hospitalized patients, we used a case–control design. We consecutively enrolled 103 DM patients with cognitive impairment and 206 age- and sex-matched DM patients without cognitive impairment in an exact 1:2 ratio (Supplementary Figure S1).

### 2.2 Assessment of diabetes mellitus and diabetic visual impairment

The presence of diabetes mellitus and diabetic retinopathy in the NHANES dataset was derived from the Diabetes Questionnaire section of the personal interview data, using variables DIQ010 and DIQ080. Individuals with missing DR status or who answered “do not know” were excluded to maintain the integrity of analyses comparing DM populations with and without DR (Figure 1). In the UKB dataset, the diagnoses of DM and DR were defined according to The International Classification of Diseases, Tenth Revision (ICD-10) codes (Supplementary Table S1). In the Wenzhou dataset, DR was diagnosed based on both fundus photography and the clinical diagnosis of endocrinologists. Two ophthalmologists, blinded to the patients’ clinical information, independently assessed 45-degree color digital images of the bilateral fundus captured using a fundus camera (VISUCAM PRO NM, VISUCAM 200).

### 2.3 Cognitive outcomes

During the 2011–2012 and 2013–2014 NHANES cycles, cognitive function tests were conducted in participants over 60 years of age at the Mobile Exam Center (MEC). The assessments consisted of the word-learning subtest of the Consortium to Establish a Registry for Alzheimer’s Disease (CERAD), Animal Fluency Test (AFT), and the Digit Symbol Substitution Test (DSST). Higher scores on these tests indicate better cognitive performance. The detailed scoring methods are provided in the Supplementary methods. Following the approach of Sun et al. (21), participants scoring below the lowest 25th percentile in each test were assigned 1 point, indicating low cognitive performance; otherwise, they were assigned 0 points. The points across the three tests were then summed to derive a composite score. Based on the overall scores, participants were categorized into a normal group (score = 0) and a cognitive function impairment (CFI) group (score > 0), making CFI the composite NHANES cognitive outcome. In the UKB dataset, the cognitive outcome was all-cause dementia, identified using ICD-10 diagnostic codes (Supplementary Table S1). In Wenzhou dataset, cognitive function was assessed by trained neuropsychologists who were blinded to the patients’ clinical data. The assessment was performed using the Chinese versions of the Mini-Mental State Examination (MMSE) during the patients’ hospitalization. Lower scores indicate poorer cognitive performance. Cognitive impairment was defined according to education-specific MMSE cutoff values: ≤17 for illiterate individuals (0 years of education), ≤20 for participants with a primary school education (1–6 years of education), and ≤24 for those with a junior high school education or above (≥7 years of education) (22). Because cognitive outcomes differed across datasets, with test-defined CFI in NHANES, ICD-10-defined all-cause dementia in UKB, and MMSE-defined cognitive impairment in the Wenzhou dataset, cross-dataset comparisons were interpreted as triangulation across related but non-equivalent indicators of adverse cognitive health, rather than as direct external validation of the same construct.

### 2.4 Dietary inflammatory index

All participants from the NHANES dataset were eligible for two 24-h dietary recall interviews. The first recall interview was conducted face-to-face at the Mobile Examination Center (MEC), and the second interview was conducted by telephone 3 to 10 days later. To improve dietary assessment accuracy and consistency, the mean of the two 24-h recalls was used in the present study. The Food Patterns Equivalents Database (FPED) was used together with NHANES dietary recall data to estimate nutrient and food-item intake. In the UKB dataset, dietary intake was assessed using the web-based, self-administered 24-h dietary questionnaire (Oxford WebQ). In the Wenzhou dataset, dietary intake was assessed using a simplified Food Frequency Questionnaire (FFQ-25), which is based on traditional Chinese dietary patterns and is suitable for investigating the eating habits of urban elderly populations in China (23, 24).

DII scores were calculated using the dietaryindex R package. According to the dietaryindex package, total energy intake was incorporated as one of the DII components in the calculation (25). The DII was designed to quantify the inflammatory potential of the overall diet, with higher scores indicating a more pro-inflammatory dietary profile (26). Different countries have created means and standard deviations of dietary parameters based on numerous databases. Briefly, for each available dietary component, a z score was calculated as: z = (individual intake − global mean intake)/global standard deviation. The z score was then converted to a centered percentile by percentile transformation, multiplication by 2, and subtraction of 1. The centered percentile for each component was multiplied by its literature-derived inflammatory effect score, and the component-specific values were summed to obtain the overall DII score.

In the present study, DII scores were calculated using 28 dietary parameters in NHANES, 28 in the UKB cohort, and 16 nutrient-derived parameters in the Wenzhou dataset. Nutrient parameters not available from a given dietary instrument or food composition database were excluded in the dataset-specific DII calculation, and no imputation was performed for unavailable components. Furthermore, we excluded participants with missing dietary data required for DII calculation (Figure 1; Supplementary Figure S1). Detailed information on the DII algorithm, dataset-specific components, and derivation of nutrient intake in each dataset is provided in Supplementary methods.

### 2.5 Assessment of covariates

Regarding demographic data, we collected data on age, sex, race and education level/years. Baseline cognitive impairment risk factors including BMI, smoking status, and alcohol consumption were also recorded through self-reported questionnaires (NHANES and UKB datasets) and hospital electronic medical record system (Wenzhou dataset). In addition, baseline data related to DM, including HbA1c, fasting plasma glucose (FPG), fasting insulin (FINS), homeostasis model assessment of insulin resistance (HOMA-IR), and diabetes duration, were collected from laboratory test results and medical records. The HOMA-IR was calculated as FPG (mmol/L) multiplied by FINS (mIU/L) and divided by 22.5. HOMA-IR is commonly used to assess insulin resistance and reflects impaired insulin sensitivity, with higher values suggesting more severe insulin resistance. The detailed definition is provided in Supplementary Table S2.

### 2.6 Statistical analysis

In NHANES dataset, continuous variables were described using the survey-weighted mean and standard error (SE), with between-group comparisons conducted using the survey-weighted linear regression. Categorical variables were characterized using the weighted percentage and confidence interval (CI), and between-group comparisons were conducted using the survey-weighted chi-square test. Because the DII was derived from the average of two 24-h dietary recalls, we used the dietary two-day sample weight (WTDR2D); for the combined 2011–2014 cycles, a 4-year dietary weight was constructed by dividing the 2-year weight by 2. The full complex survey design was incorporated using SDMVSTRA as the stratification variable and SDMVPSU as the primary sampling unit variable. Because NHANES uses a complex, multistage probability sampling design, weighted multivariable-adjusted Poisson regression models were used to estimate prevalence ratios (PRs) and 95% confidence intervals (CIs) for the associations of DM, DR, DII, and HbA1c with cognitive outcomes. All models were adjusted for age, sex, race, educational level, BMI, smoking status, and drinking status. Survey-weighted descriptive statistics and regression models were implemented in R (version 4.2.0) using the survey package. In UKB and Wenzhou datasets, continuous variables were presented as mean (standard deviation) or median (interquartile range), depending on the data distribution. Categorical variables were shown as frequencies (percentages). Student’s t-test was used for the comparison of normally distributed variables, while the Mann–Whitney U test was applied to continuous variables with asymmetrical distribution. The Chi-square test was used to compare categorical variables.

In the UKB dataset, participants with DM were matched to participants without DM at a 1:2 ratio using propensity score matching to reduce baseline imbalance and improve comparability between groups. The matching criteria included age, sex, race, educational years, and BMI. Matching was performed using the nearest-neighbor method with a caliper of 0.1. Covariate balance before and after matching was assessed using standardized mean differences (SMDs), with an absolute SMD < 0.1 considered indicative of acceptable balance. Density plots of the propensity score distributions and Love plots of the absolute SMDs were generated to visually assess common support and covariate balance after matching. In the propensity score-matched dataset, a cluster-robust Cox proportional hazards regression model was fitted, with matched subclasses specified as clusters and robust sandwich variance estimation used to account for the matched design. Given the exact 1:2 matched case–control design of the Wenzhou dataset, standard conditional logistic regression was used as the primary analytical approach to evaluate the associations between candidate factors and cognitive impairment. As a sensitivity analysis for potential overfitting in the Wenzhou dataset, Firth penalized conditional logistic regression was also performed.

Exploratory mediation analyses were performed in all three datasets under prespecified models involving DII. In the NHANES dataset, DM and DR were treated as antecedent variables, the dietary index as a potential intermediate variable, and cognitive function as the outcome. Because these measures were obtained within the same survey cycle, the temporal ordering could not be definitively established. Therefore, the mediation analysis in NHANES was considered exploratory and hypothesis-generating rather than evidence of causal mediation. In the UKB dataset, exploratory mediation analysis was conducted under a more plausible temporal sequence, with DM specified as the exposure, baseline DII and HbA1c as the candidate intermediate variable, and subsequent all-cause dementia as the outcome. To preserve the hypothesized temporal sequence, participants with dementia diagnosed before baseline, dementia diagnosed before DM, or DM diagnosed after baseline were excluded. In the final analytic sample, the date of DM diagnosis preceded the baseline assessment date by a median of 3.7 years (IQR, 1.6–6.6), whereas the date of all-cause dementia diagnosis occurred a median of 10.4 years (IQR, 8.1–11.9) after baseline. Therefore, the temporal ordering of the UKB dataset was specified as diabetes → baseline dietary index → subsequent all-cause dementia, which provides a reasonable epidemiological basis for conducting exploratory mediation analysis.

In the Wenzhou dataset, exploratory mediation analysis treated DR was treated as the exposure, the dietary index derived from the FFQ-25 as the mediator, and cognitive impairment as the outcome. In this dataset, all participants had established diabetes before hospital admission. Fundus photography was routinely performed on the day of admission to determine DR status. Dietary intake was assessed after admission using the FFQ-25, which captured habitual dietary intake over the previous 6 months, and cognitive function was evaluated near discharge. Because DR was considered a pre-existing chronic microvascular complication rather than a newly developed condition at the time of fundus examination, and the FFQ-25 reflected dietary exposure during the 6 months preceding cognitive assessment, the temporal ordering was specified as DR → dietary index → cognitive impairment. This ordering allowed exploratory analysis under a more plausible temporal sequence but did not support causal inference. For all mediation analyses, indirect, direct, and total effects were estimated, and the proportion mediated was calculated as (indirect effect / total effect) × 100. Bias-corrected bootstrap confidence intervals were obtained using 1,000 resamples.

Restricted cubic spline analyses were prespecified and conducted within survey-weighted logistic regression models to assess the association between DII and CFI group. In the primary analysis, 5 knots were placed at the 5th, 27.5th, 50th, 72.5th, and 95th percentiles of the DII distribution. The median DII value was used as the reference point, and adjusted odds ratios (ORs) with 95% confidence intervals (CIs) were estimated across the observed DII range. Sensitivity analyses were performed using 4-knot and 6-knot specifications. Overall and nonlinear associations were evaluated using design-based Wald tests. To further explore whether a threshold-like change in slope could be estimated, complementary exploratory survey-weighted segmented logistic regression analyses were also performed in NHANES (Supplementary methods).

The subgroup analyses, mediation analyses, and restricted cubic spline analyses were considered secondary or exploratory analyses. All statistical analyses were performed using R version 4.2.0 (R Foundation for Statistical Computing, Vienna, Austria). All *p* values were two-sided, and *p* < 0.05 was considered statistically significant.

## 3 Results

### 3.1 Characteristics of all participants

Overall, 2,524 and 25,427 participants were included from NHANES and the UKB dataset, respectively (Figure 1). To further analyze the impact of DR on cognition, we subsequently excluded participants lacking information on DR. The NHANES sample consisted of 505 DM participants without DR and 123 DM participants with DR. Following propensity score matching in the UKB dataset, 734 participants with diabetes and 1,467 participants without diabetes remained in the final matched sample.

The characteristics of participants with and without DM in NHANES are shown in Table 1. The prevalence of the composite outcome CFI was significantly higher in participants with DM than in those without DM (42.8% vs. 27.7%, *p* < 0.001). Higher prevalences of low cognitive performance on CERAD, AFT, and DSST were also observed (CERAD: 22.5% vs. 16.1%, *p* = 0.010; AFT: 21.5% vs. 12.3%, *p* < 0.001; DSST: 22.5% vs. 10.1%, *p* < 0.001). Participants with DM also had significantly higher HbA1c, BMI, and DII scores than those without DM (all *p* < 0.05). Significant differences were also observed for sex, race, educational level, smoking status, and drinking status, whereas no significant differences were found for age and marital status. Similar results were observed in the UKB dataset. Before PSM, participants with DM had a significantly higher rate of all-cause dementia than those without DM (4.7% vs. 2.0%, *p* < 0.001), along with higher DII scores, HbA1c levels and BMI. Significant differences were also observed in age, sex, Townsend deprivation index, race, educational years, and drinking status. After PSM, the distributions of both categorical and continuous matching variables became highly comparable between participants with and without DM, and covariate balance was markedly improved, with all standardized mean differences for the matching variables below 0.1, indicating adequate matching performance (Table 2). Post-PSM balance diagnostics further confirmed the effectiveness of matching, as evidenced by the closely aligned covariate distributions and substantially reduced absolute standardized mean differences in Supplementary Figure S2. In the matched dataset, participants with DM still had a significantly higher rate of all-cause dementia (4.6% vs. 1.9%, *p* < 0.001), together with higher DII scores, HbA1c levels.

**Table 1:** Characteristics of the participants in NHANES dataset.

Variables	All participants		*p*-value	DM participants with DR data		*p*-value
	Absence of DM Mean (SE) or % (95% CI)	Presence of DM Mean (SE) or % (95% CI)		Absence of DR Mean (SE) or % (95% CI)	Presence of DR Mean (SE) or % (95% CI)	
N^a^	1892	632		505	123	
Frequency (weighted)^b^	40,608,678	10,525,207		8,809,710	1,681,362	
Age (years)	69.2 (0.3)	69.4 (0.3)	0.555	69.3 (0.3)	70.2 (0.8)	0.281
Age stratification			0.653			0.349
60–70	61.1 (57.4, 64.7)	59.9 (55.8, 63.9)		60.9 (56.2, 65.4)	55.1 (43.5, 66.1)	
70–80	38.9 (35.3, 42.6)	40.1 (36.1, 44.2)		39.1 (34.6, 43.8)	44.9 (33.9, 56.5)	
Gender (male)	44.2 (42.0, 46.5)	51.5 (45.0, 57.9)	0.047	51.2 (44.1, 58.3)	52.1 (39.7, 64.2)	0.894
Race/ethnicity			<0.001			0.014
Mexican American	2.8 (1.7, 4.4)	6.5 (3.6, 11.7)		5.9 (3.0, 11.3)	9.5 (5.0, 17.3)	
Other Hispanic	3.6 (2.3, 5.6)	4.5 (3.0, 6.6)		3.9 (2.4, 6.4)	7.3 (3.6, 14.1)	
Non-Hispanic White	81.8 (77.9, 85.2)	69.3 (62.5, 75.3)		71.1 (64.1, 77.2)	59.6 (48.4, 69.9)	
Non-Hispanic Black	6.9 (5.1, 9.3)	12.8 (9.1, 17.8)		11.6 (8.1, 16.3)	19.4 (12.6, 28.7)	
Non-Hispanic Asian	3.6 (2.7, 4.8)	4.1 (2.5, 6.7)		4.2 (2.6, 6.8)	3.6 (1.1, 11.6)	
Other Race	1.2 (0.7, 2.2)	2.9 (1.3, 6.1)		3.3 (1.5, 7.1)	0.6 (0.1, 4.7)	
Education level			<0.001			0.006
Below High school	13.9 (11.0, 17.5)	25.6 (20.6, 31.2)		23.5 (18.6, 29.4)	35.9 (26.6, 46.4)	
High school	21.2 (18.3, 24.6)	25.9 (21.4, 31.1)		25.1 (20.4, 30.4)	29.7 (19.7, 42.0)	
Above High school	64.8 (60.9, 68.5)	48.5 (42.2, 54.8)		51.4 (44.6, 58.1)	34.4 (25.5, 44.6)	
HbA1c, %	5.6 (0.1)	7.1 (0.1)	<0.001	7.0 (0.1)	7.9 (0.2)	0.057
BMI, kg/m^2^	28.3 (0.2)	31.7 (0.3)	<0.001	31.7 (0.4)	31.6 (0.6)	0.927
Smoking status	48.5 (45.0, 52.0)	56.8 (52.2, 61.4)	0.001	56.9 (50.9, 62.7)	57.2 (45.2, 68.4)	0.966
Drinking status	73.5 (70.0, 76.7)	68.0 (63.7, 72.0)	0.011	69.1 (63.8, 74.0)	62.4 (49.3, 73.9)	0.335
Marital status	62.6 (59.5, 65.6)	61.0 (57.4, 64.4)	0.455	62.2 (58.5, 65.9)	53.6 (44.7, 62.3)	0.057
CFI	27.7 (24.1, 31.6)	42.8 (37.7, 48.0)	<0.001	38.3 (33.5, 43.4)	65.4 (52.4, 76.5)	<0.001
CERAD	16.1 (13.2, 19.5)	22.5 (18.2, 27.5)	0.010	20.1 (16.0, 25.0)	34.4 (24.6, 45.6)	0.001
AFT	12.3 (10.5, 14.4)	21.5 (17.3, 26.4)	0.001	20.5 (15.8, 26.2)	27.1 (18.1, 38.5)	0.234
DSST	10.1 (8.1, 12.4)	22.5 (16.9, 29.2)	<0.001	18.9 (13.4, 25.9)	41.2 (30.1, 53.2)	<0.001
Dietary indices						
DII score	1.3 (0.1)	1.8 (0.1)	<0.001	1.7 (0.1)	2.2 (0.2)	0.010

a The unweighted number of cases.

b All cases are weighted to be nationally representative.

Continuous variables are presented as weighted means (weighted standard errors) and categorical variables as weighted percentages (95% CI).

AFT, Animal Fluency Test; BMI, Body Mass Index; CERAD, Consortium to Establish a Registry for Alzheimer’s Disease; CFI, Cognitive Function Impairment; CI, Confidence Interval; DII, Dietary Inflammatory Index; DM, Diabetes Mellitus; DSST, Digit Symbol Substitution Test.

**Table 2:** Characteristics of the participants in UKB dataset.

Variables	Before PSM		*p*-value	After PSM		*p*-value	SMD
	Absence of DM Mean (SD) or *n* (%)	Presence of DM Mean (SD) or *n* (%)		Absence of DM Mean (SD) or *n* (%)	Presence of DM Mean (SD) or *n* (%)		
*N*	24,685	742		1,467	734		
Age (years)	63.9 (2.7)	64.6 (2.8)	<0.001	64.6 (2.8)	64.6 (2.8)	0.890	0.006
Gender (male, %)	11,377 (46.1%)	489 (65.9%)	<0.001	971 (66.2%)	483 (65.8%)	0.857	−0.007
Townsend Deprivation Index	−2.3 (−3.6 to −0.1)	−1.5 (−3.2–1.0)	<0.001	−2.2 (−3.6 to −0.1)	−1.5 (−3.2–1.0)	<0.001	NA
Race/ethnicity			<0.001			0.243	
White	24,103 (97.6%)	675 (91.0%)		1,369 (93.3%)	675 (92.0%)		−0.045
Non-White	582 (2.4%)	67 (9.0%)		98 (6.7%)	59 (8.0%)		0.045
Education years	13.0 (10.0–20.0)	10.0 (7.0–18.0)	<0.001	10.0 (7.0–18.0)	10.0 (7.0–18.0)	0.909	0.007
HbA1c (mmol/mol)	35.6 (33.5–37.9)	49.7 (43.4–57.7)	<0.001	36.0 (33.8–38.3)	49.8 (43.3–57.7)	<0.001	NA
BMI, kg/m^2^			<0.001			0.833	NA
< 25	8,690 (35.2%)	88 (11.9%)		164 (11.2%)	88 (12.0%)		0.025
25–30	11,107 (45.0%)	288 (38.8%)		574 (39.1%)	288 (39.2%)		0.003
≥ 30	4,888 (19.8%)	366 (49.3%)		729 (49.7%)	358 (48.8%)		−0.019
Smoking status	1,522 (6.2%)	46 (6.2%)	0.970	82 (5.6%)	45 (6.1%)	0.608	NA
Drinking status	22,967 (93.0%)	650 (87.6%)	<0.001	1,329 (90.6%)	645 (87.9%)	0.048	NA
DII score	−0.3 (−1.8–1.2)	0.0 (−1.5–1.6)	<0.001	−0.2 (−1.9–1.3)	0.0 (−1.5–1.6)	0.013	NA
All-cause dementia	492 (2.0%)	35 (4.7%)	<0.001	28 (1.9%)	34 (4.6%)	<0.001	NA

Continuous variables are presented as means (standard deviation) or medians (interquartile range), and categorical variables as number (percentages). BMI, Body Mass Index; CI, Confidence Interval; DII, Dietary Inflammatory Index; DM, Diabetes Mellitus; SMD, standardized mean differences.

To compare different severity levels of DM, we included DM participants with DR data available for the comparison between DR and non-DR individuals. In NHANES (Table 1), participants with DR had a significantly higher prevalence of the composite outcome CFI than those without DR (65.4% vs. 38.3%, *p* < 0.001). This difference was mainly reflected in cognitive impairment defined by CERAD and DSST scores (CERAD: 34.4% vs. 20.1%, *p* = 0.001; DSST: 41.2% vs. 18.9%, *p* < 0.001), whereas the proportion defined by AFT score did not differ significantly between the two groups (27.1% vs. 20.5%, *p* = 0.234). In addition, participants with DR had significantly higher DII scores (2.2 vs. 1.7, *p* = 0.010). Significant differences were also observed in race and educational level (both *p* < 0.05), whereas age, sex, HbA1c, BMI, smoking status, drinking status, and marital status were comparable between the two groups. In the case–control design of the Wenzhou dataset (Table 3), among all DM participants, those with cognitive impairment had significantly fewer years of education [3.0 (0.0–8.0) vs. 5.0 (2.0–8.0), *p* = 0.014], lower BMI (23.3 ± 3.1 vs. 24.1 ± 3.1 kg/m^2^, *p* = 0.038), a markedly higher proportion of DR (56.3% vs. 21.8%, *p* < 0.001), and higher DII scores [2.2 (1.5–2.5) vs. 1.6 (1.2–2.0), *p* < 0.001] than those without cognitive impairment. By contrast, no significant differences were found in age, sex, educational degree, HbA1c, FPG, FINS, HOMA-IR, diabetes duration, smoking status, or drinking status (all *p* > 0.05). To evaluate potential selection bias due to complete-case analysis, we compared included and excluded participants in the NHANES dataset (Supplementary Table S3). Excluded participants had higher HbA1c levels and differed in race/ethnicity and drinking status, whereas age, sex, education level, BMI, smoking status, DM, and DR were comparable between the two groups.

**Table 3:** Characteristics of all DM participants in Wenzhou dataset.

Variables	Absence of cognitive impairment	Presence of cognitive impairment	*p*-value
*N*	206	103	
Age (years)	66.0 (4.4)	66.0 (4.4)	1.000
Gender (male, %)	104 (50.5%)	52 (50.5%)	1.000
Education degree			0.374
Illiterate	42 (20.4%)	26 (25.2%)	
Primary school	80 (38.8%)	43 (41.7%)	
Secondary school or above	84 (40.8%)	34 (33.0%)	
Education years	5.0 (2.0–8.0)	3.0 (0.2–8.0)	0.014
BMI, kg/m^2^	24.1 (3.1)	23.3 (3.1)	0.038
HbA1c, %	9.3 (8.0–10.7)	9.7 (8.3–11.2)	0.096
FPG (mmol/L)	8.2 (6.8–10.0)	8.6 (6.7–11.2)	0.167
FINS (pmol/L)	43.3 (29.0–69.8)	39.6 (24.8–59.6)	0.105
HOMA-IR	2.8 (1.8–4.5)	2.6 (1.5–4.2)	0.284
Diabetes duration, years			0.803
≤ 5	37 (18.0%)	21 (20.4%)	
6–10	53 (25.7%)	30 (29.1%)	
11–20	78 (37.9%)	34 (33.0%)	
> 20	38 (18.4%)	18 (17.5%)	
DR	45 (21.8%)	58 (56.3%)	<0.001
Smoking status	57 (27.7%)	36 (35.0%)	0.188
Drinking status	53 (25.7%)	26 (25.2%)	0.927
DII score	1.6 (1.2–2.0)	2.2 (1.5–2.5)	<0.001
MMSE score	25.0 (22.0–27.0)	18.0 (16.0–21.0)	<0.001

Continuous variables are presented as means (standard deviation) or medians (interquartile range), and categorical variables as number (percentages). BMI, Body Mass Index; DII, Dietary Inflammatory Index; DM, Diabetes Mellitus; DR, diabetic retinopathy; FPG, Fasting plasma glucose; FINS, Fasting insulin; HOMA-IR, Homeostasis model assessment of insulin resistance; MMSE, Mini-Mental State Examination.

### 3.2 Associations of DII, DM, and DR with low cognitive performance

The associations between DII, DM, HbA1c and different measures of low cognitive performance among NHANES participants are shown in Table 4. In the overall NHANES sample, weighted multivariable-adjusted Poisson regression analyses showed that DM was significantly associated with a higher prevalence of low cognitive performance on the AFT (PR = 1.399, 95% CI: 1.097–1.785, *p* = 0.014), DSST (PR = 1.571, 95% CI: 1.222–2.018, *p* = 0.002), and CFI (PR = 1.260, 95% CI: 1.083–1.466, *p* = 0.008), but not on the CERAD test (PR = 1.219, 95% CI: 0.960–1.549, *p* = 0.121). Higher DII was consistently associated with a greater prevalence of low cognitive performance, including CERAD (PR = 1.228, 95% CI: 1.125–1.341, *p* < 0.001), AFT (PR = 1.253, 95% CI: 1.157–1.357, *p* < 0.001), DSST (PR = 1.236, 95% CI: 1.136–1.343, p < 0.001), and CFI (PR = 1.192, 95% CI: 1.127–1.261, *p* < 0.001). Higher HbA1c was also significantly associated with low cognitive performance on the CERAD test (PR = 1.110, 95% CI: 1.023–1.205, p = 0.014), DSST (PR = 1.150, 95% CI: 1.074–1.231, *p* = 0.001), and CFI (PR = 1.079, 95% CI: 1.016–1.147, *p* = 0.023), whereas no significant association was observed for AFT (PR = 1.105, 95% CI: 0.999–1.221, *p* = 0.051). We then compared participants with and without DR among NHANES participants with DM (Table 5). Among participants with DM, DR was significantly associated with a higher prevalence of low cognitive performance on the CERAD test (PR = 1.382, 95% CI: 1.024–1.866, *p* = 0.049), DSST (PR = 1.690, 95% CI: 1.201–2.378, *p* = 0.007), and CFI (PR = 1.414, 95% CI: 1.167–1.713, p = 0.001), but not on the AFT (PR = 1.131, 95% CI: 0.729–1.755, *p* = 0.562). Higher DII was significantly associated with low cognitive performance on the AFT (PR = 1.421, 95% CI: 1.226–1.646, *p* < 0.001), DSST (PR = 1.219, 95% CI: 1.046–1.422, *p* = 0.014), and CFI (PR = 1.186, 95% CI: 1.106–1.272, *p* < 0.001), whereas its association with CERAD did not reach statistical significance (PR = 1.146, 95% CI: 0.992–1.324, *p* = 0.062). HbA1c was not significantly associated with any of the cognitive measures in this subgroup after multivariable adjustment.

**Table 4:** Weighted multivariable-adjusted Poisson regression analyses of the associations of DM, DII, and HbA1c with low cognitive performance among NHANES participants (*N* = 2,524).

Variables	CERAD test	AFT	DSST	CFI
	PR (95%CI)	PR (95%CI)	PR (95%CI)	PR (95%CI)
DM	1.219 (0.960–1.549)	1.399 (1.097–1.785)^*^	1.571 (1.222–2.018)^**^	1.260 (1.083–1.466)^**^
DII	1.228 (1.125–1.341)^***^	1.253 (1.157–1.357)^***^	1.236 (1.136–1.343)^***^	1.192 (1.127–1.261)^***^
HbA1c	1.110 (1.023–1.205)^*^	1.105 (0.999–1.221)	1.150 (1.074–1.231)^**^	1.079 (1.016–1.147)^*^

AFT, Animal Fluency Test; CERAD, Consortium to Establish a Registry for Alzheimer’s Disease; CFI, Cognitive Function Impairment; CI, Confidence Interval; DII, Dietary Inflammatory Index; DM, Diabetes Mellitus; DSST, Digit Symbol Substitution Test. Adjusted for age, sex, race, educational level, BMI, smoking status, drinking status. *p < 0.05, **p < 0.01 and ***p < 0.001.

**Table 5:** Weighted multivariable-adjusted Poisson regression analyses of the associations of DR, DII, and HbA1c with low cognitive performance among NHANES participants with DM (*N* = 628).

Variables	CERAD test	AFT	DSST	CFI
	PR (95%CI)	PR (95%CI)	PR (95%CI)	PR (95%CI)
DR	1.382 (1.024–1.866)^*^	1.131 (0.729–1.755)	1.690 (1.201–2.378)^**^	1.414 (1.167–1.713)^**^
DII	1.146 (0.992–1.324)	1.421 (1.226–1.646)^***^	1.219 (1.046–1.422)^*^	1.186 (1.106–1.272)^***^
HbA1c	1.082 (0.988–1.185)	1.029 (0.934–1.133)	1.086 (0.997–1.183)	1.022 (0.956–1.093)

AFT, Animal Fluency Test; CERAD, Consortium to Establish a Registry for Alzheimer’s Disease; CFI, Cognitive Function Impairment; CI, Confidence Interval; DII, Dietary Inflammatory Index; DR, diabetic retinopathy; DSST, Digit Symbol Substitution Test. Adjusted for age, sex, race, educational level, BMI, smoking status, drinking status. *p < 0.05, **p < 0.01 and ***p < 0.001.

In two additional datasets with different cognitive outcome definitions, we further examined whether a similar association pattern could be observed using regression models appropriate for each dataset. In the UKB dataset, the fully adjusted robust Cox proportional hazards model showed that DM, higher DII, and higher HbA1c were independently associated with a higher hazard of all-cause dementia (DM: HR = 2.711, 95% CI: 1.641–4.478, *p* < 0.001; DII: HR = 1.201, 95% CI: 1.072–1.346, *p* = 0.002; HbA1c: HR = 1.027, 95% CI: 1.012–1.042, *p* < 0.001) (Supplementary Table S4). In the Wenzhou dataset, the fully adjusted conditional logistic regression model showed that DR and higher DII were significantly associated with greater odds of cognitive impairment (DR: OR = 7.555, 95% CI: 3.706–15.401, *p* < 0.001; DII: OR = 12.642, 95% CI: 3.987–40.080, *p* < 0.001), whereas HbA1c and HOMA-IR were not significantly associated with cognitive impairment (HbA1c: OR = 1.048, 95% CI: 0.934–1.177, *p* = 0.425; HOMA-IR: OR = 0.953, 95% CI: 0.842–1.078, *p* = 0.440) (Supplementary Table S5). Results from Firth penalized conditional logistic regression were materially similar to those from the standard conditional logistic regression, suggesting that the effect estimates from the standard model in the Wenzhou dataset were reasonably stable (Supplementary Table S6). These findings further supported the associations of DM-related factors and DII with adverse cognitive outcomes.

### 3.3 Mediation analysis

In the NHANES dataset, two exploratory mediation analyses were performed. In the overall NHANES sample, DII showed significant indirect effects in the association between DM and low cognitive performance, including CFI (Indirect effect = 0.012, 95%CI = 0.006–0.019; mediated proportion = 15.0%), CERAD (Indirect effect = 0.009, 95%CI: = 0.004–0.015; mediated proportion = 15.4%), AFT (Indirect effect = 0.010, 95%CI = 0.005–0.016; mediated proportion = 18.7%), and DSST (Indirect effect = 0.008, 95% CI = 0.003–0.013; mediated proportion = 11.9%). In contrast, HbA1c did not show significant indirect effects for CFI, CERAD, or AFT, but showed a significant indirect effect for DSST (Indirect effect = 0.027, 95%CI = 0.005–0.049; mediated proportion = 43.1%) (Supplementary Table S7). Among NHANES participants with DM, only DII showed a statistically significant indirect effect for CFI [0.010 (95% CI: 0.000–0.022), mediated proportion: 8.0%]. HbA1c did not show significant indirect effects for any cognitive outcome in this subgroup (Supplementary Table S8). Given the cross-sectional nature of NHANES and the contemporaneous assessment of the intermediate variables and cognitive outcomes within the same survey cycle, these findings should be interpreted as exploratory and hypothesis-generating rather than as definitive evidence of causal mediation.

**Figure 2:**
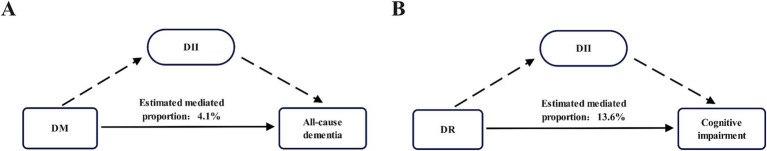
Exploratory mediation analyses of the dietary inflammatory index under the assumed pathway framework in the UKB and Wenzhou datasets. **(A)** Exploratory mediation analysis of DII in the association between DM and all-cause dementia in the UKB dataset under the assumed temporal ordering. This model is adjusted for age, sex, race, educational years, BMI, smoking status, and drinking status. **(B)** Exploratory mediation analysis of DII in the association between DR and cognitive impairment in the Wenzhou dataset under the hypothesized temporal sequence. This model is adjusted for educational years, BMI, smoking status, drinking status, and diabetes duration. Because these estimates were obtained from observational data, the pathways shown should be interpreted under the specified framework rather than as definitive evidence of causal mediation.

We then examined whether similar indirect-effect patterns could also be observed in two additional datasets. In the UKB dataset, exploratory mediation analysis showed a significant indirect effect of DII in the association between DM and subsequent all-cause dementia. The indirect effect of DII was 0.001 (95%CI = 0.000–0.003), accounting for 4.1% of the model-based total effect, whereas HbA1c did not show a significant indirect effect (Figure 2; Supplementary Table S9). In the Wenzhou dataset, exploratory mediation analysis also showed a significant indirect effect of DII in the association between DR and cognitive impairment, with an indirect effect of 0.496 (95%CI = 0.162–1.042), corresponding to 13.6% of the model-based total effect, whereas neither HbA1c nor HOMA-IR showed significant indirect effects (Figure 2; Supplementary Table S10). Taken together, these analyses showed a broadly similar indirect-effect pattern for DII across related but non-equivalent cognitive outcomes, whereas the corresponding results for HbA1c were not consistent.

**Figure 3:**
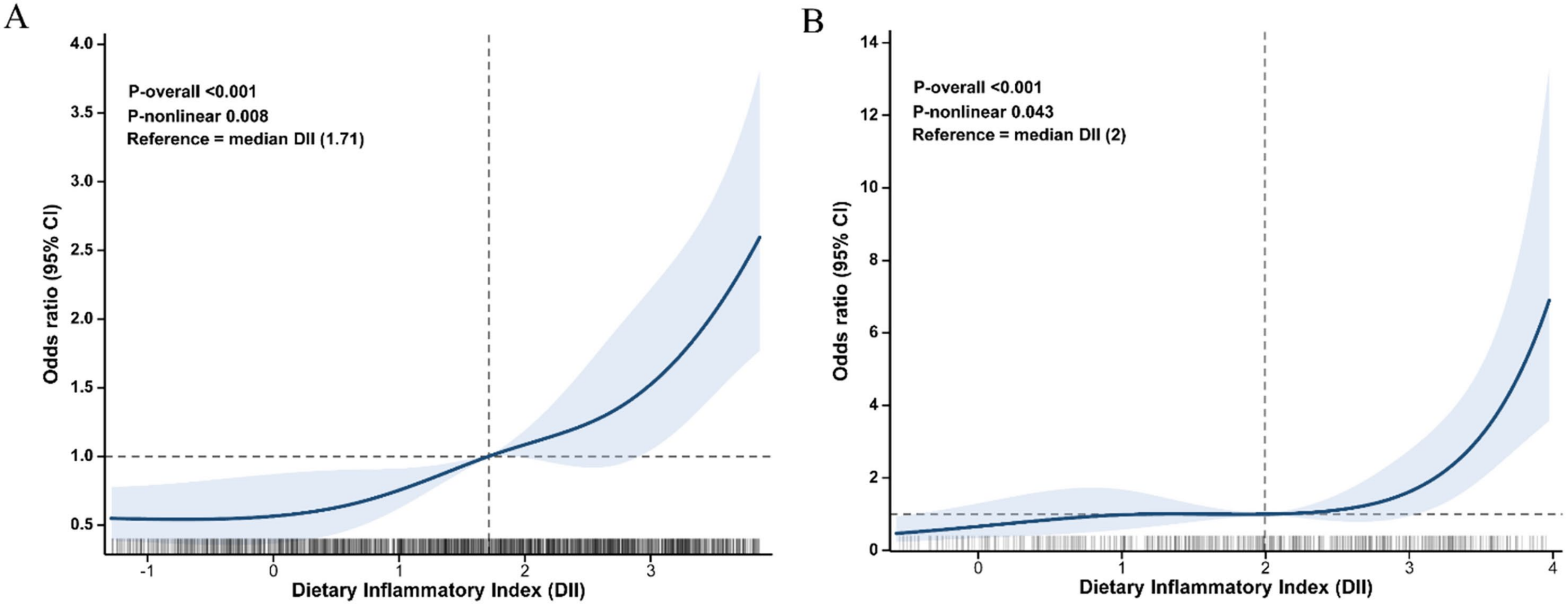
Restricted cubic spline analysis of the association between DII and CFI group. Adjusted odds ratios (ORs) with 95% confidence intervals (CIs) were estimated from survey-weighted multivariable logistic regression models using 5-knot restricted cubic splines, with knots placed at the 5th, 27.5th, 50th, 72.5th, and 95th percentiles of the DII distribution and the median DII as the reference. **(A)** Association between DII and CFI group in all participants. **(B)** Association between DII and CFI group in diabetes participants with available data on DR.

In the overall NHANES population, the RCS analysis demonstrated a significant overall association between DII and CFI (P-overall < 0.001) and significant evidence of nonlinearity (P-nonlinearity = 0.008) (Figure 3). Using the median DII as the reference value, DII levels below the reference were associated with lower odds of CFI, whereas DII levels above the reference were associated with higher odds of CFI. Overall, the spline curve suggested a gradual nonlinear positive association, with the odds of CFI increasing more clearly at higher DII levels rather than showing an abrupt step-like change. In sensitivity analyses, the results were materially unchanged when 4 knots (P-overall < 0.001; P-nonlinearity = 0.005) and 6 knots (P-overall < 0.001; P-nonlinearity = 0.015) were used, supporting the robustness of the nonlinear association (Supplementary Figure S3). Similar findings were observed among participants with diabetes. Taken together, the RCS analyses in both the overall population and the diabetes subgroup suggested a consistent nonlinear positive association between DII and the odds of CFI. Exploratory survey-weighted segmented analyses yielded similar evidence of a steeper association at higher DII levels, but did not support a clear or robust threshold (Supplementary Table S11).

## 4 Discussion

In this observational study using three complementary datasets, the primary analyses in NHANES showed that DM and DR were both associated with adverse cognitive outcomes in older adults, and that higher DII was consistently associated with poorer cognitive performance. Similar association patterns were further observed in the UKB and Wenzhou datasets, despite differences in cognitive outcome definitions and sampling frameworks. Exploratory mediation analyses suggest that dietary inflammatory profile may be relevant to the observed associations of DM and DR with adverse cognitive outcomes, but they do not establish causality.

In both the NHANES and UKB datasets, participants with DM consistently exhibited a more adverse metabolic and vascular profile than those without DM, including higher HbA1c levels and BMI. These findings are broadly consistent with previous evidence (27, 28). Significant differences were also consistently observed in sex, race, and educational attainment, suggesting that DM in these populations was patterned not only by metabolic risk but also by broader demographic and social factors (29, 30). Importantly, DM was associated with worse cognitive outcomes across datasets. In NHANES, participants with DM had a higher prevalence of low cognitive performance, whereas in UKB, DM was associated with a higher hazard of all-cause dementia. In addition, DII showed a broadly similar exploratory association pattern with adverse cognitive outcomes across NHANES, UKB, and Wenzhou. Together, these findings suggest that DM is associated with an increased cognitive burden and that a more pro-inflammatory dietary profile may be one factor relevant to diabetes-related cognitive vulnerability (31, 32). These findings are consistent with previous studies suggesting that the association between DM and cognitive dysfunction is multifactorial and may involve diabetes duration (33), poor glycemic control (31), microvascular complications (34), and diabetes-related structural and neurodegenerative brain changes (35). Notably, the persistence of a higher dementia rate after matching in UKB suggests that the excess cognitive burden associated with DM is unlikely to be explained solely by baseline demographic imbalance.

To further explore heterogeneity within the diabetic population, we used DR as an indicator of greater diabetes severity rather than considering DM as a homogeneous condition. DR is a common microvascular complication of longstanding or poorly controlled diabetes and may reflect cumulative metabolic and vascular damage (31, 34). In our study, diabetic participants with DR had a greater cognitive burden than those without DR, and this pattern was observed in both the NHANES and Wenzhou datasets. These findings support the view that more severe diabetes, as represented by DR, is associated with a greater cognitive burden than DM alone. The association between DR and cognitive dysfunction is biologically plausible. The retina and brain share similar embryological origins and microvascular characteristics, and retinal abnormalities have been proposed as accessible markers of broader cerebral microvascular and neurodegenerative injury (36). Retinal neurovascular diseases are reported to precede and even predict the development of cognitive decline (37). Chronic hyperglycemia has been found to promote oxidative stress, inflammation, endothelial dysfunction, and barrier disruption, thereby contributing to both retinal injury and central nervous system damage (38–41).

In the NHANES dataset, mediation analysis was exploratory because exposure, mediator, and cognitive outcomes were obtained within the same survey cycle, and the temporal ordering therefore could not be definitively established. In contrast, the temporal sequence was more plausible in the UKB dataset and reasonably specified in the Wenzhou dataset. Rather than direct external validation of the same construct, these two datasets provided triangulation across related but non-equivalent cognitive outcomes and clinical settings. Specifically, the UKB dataset examined the exploratory indirect-effect pattern of DII in the association between DM and subsequent all-cause dementia, whereas the Wenzhou dataset examined the exploratory indirect-effect pattern of DII in the association between DR and cognitive impairment. Across these analyses, DII showed a more consistent indirect-effect pattern than HbA1c, suggesting that dietary inflammatory profile may be related to the observed associations of DM/DR with adverse cognitive outcomes; however, these findings should be interpreted as exploratory and non-causal. At the same time, the mediated proportions differed substantially across datasets and should not be interpreted as confirmatory consistency. In particular, the larger estimate in the Wenzhou dataset may reflect its smaller sample size and dataset-specific measurement framework. Therefore, these findings are better interpreted as supportive triangulating evidence for a similar indirect-effect pattern involving DII, rather than validation of a common mediation magnitude. Biological plausibility for this pattern also exists. The DII was developed to capture the inflammatory potential of diet and has been linked to inflammatory biomarkers including IL-1β, IL-4, IL-6, IL-10, TNF-α, and C-reactive protein (42). Several previous studies have verified the performance of the DII score for predicting chronic inflammation, i.e., by detecting increases in inflammatory marker concentrations in plasma (43–46). This is relevant to diabetic cognitive dysfunction because inflammation and oxidative stress are closely involved in hyperglycemia-related neuronal injury (47). Hyperglycemia can reduce antioxidant defenses, increase inflammatory signaling, and promote persistent cellular stress, thereby amplifying oxidative damage and neurodegenerative processes that may contribute to cognitive decline (48, 49). In addition, previous studies have implicated inflammation in the pathogenesis of DR, suggesting that inflammatory processes may help explain the observed indirect-effect pattern involving DII (13, 50, 51); however, these mechanisms were not directly assessed in the present study.

From a clinical perspective, our findings highlight the potential relevance of dietary inflammatory profile, as reflected by DII, in the relationship of DM and DR with cognitive outcomes. The observation that DII showed a more consistent indirect-effect signal than HbA1c across datasets suggests that dietary inflammatory status may provide additional information beyond conventional glycemic indicators when evaluating cognitive vulnerability in diabetes. These findings suggest that dietary inflammatory assessment may be useful to consider in future research on diabetic cognitive dysfunction (52–54). However, prospective studies together with randomized dietary trials are needed to establish causality and potential clinical benefit.

Several limitations should be acknowledged. First, the NHANES analyses were cross-sectional; therefore, the temporal ordering among exposure, the intermediate variable, and cognitive outcome could not be definitively established, and the NHANES mediation findings should be interpreted as exploratory rather than mechanistic. Second, direct comparability across datasets was limited because diet, DR, and cognitive outcomes were assessed differently in NHANES, UKB, and Wenzhou. In NHANES, dietary intake and DR status were based partly on self-report/interview data, which may have introduced recall and misclassification bias. Third, because complete-case analysis was used, some selection bias may remain, although included and excluded participants were broadly similar for several major characteristics. Fourth, residual confounding cannot be excluded, as some clinically relevant variables were unavailable or not uniformly captured across datasets. Fifth, the subgroup analyses, mediation analyses, and restricted cubic spline analyses were exploratory and should be interpreted cautiously because multiple comparisons were performed. Sixth, some outcomes were not rare in this study. As a result, some effect estimates may appear larger than the true strength of association, especially the odds ratios from the Wenzhou case–control analyses. Finally, the NHANES cognitive outcomes were based on an operational, sample-dependent definition, which may be influenced by educational and cultural/language factors, although education level was adjusted for in all NHANES models.

## 5 Conclusion

In conclusion, across three complementary observational datasets from three countries, higher DII scores were associated with adverse cognitive outcomes in older adults with DM/DR. We identified a broadly similar exploratory indirect-effect pattern involving DII in the associations of DM/DR with adverse cognitive outcomes in older adults. These findings should be interpreted as exploratory and non-causal, and further prospective studies and randomized controlled trials are needed to clarify causality and potential clinical benefit.

## Data Availability

The raw data supporting the conclusions of this article will be made available by the authors, without undue reservation.

## References

[1] Duncan BB Magliano DJ Boyko EJ. IDF Diabetes Atlas 11th edition 2025: global prevalence and projections for 2050. Nephrology Dialysis Transplantation. (2026) 41:7–9. doi: 10.1093/ndt/gfaf177 40874767

[2] Jacobsen LA Mather K Lee M Kent M. America's aging population. Population Bulletin Population Reference Bureau. (2011) 66:1–15. Available online at: https://www.prb.org/wp-content/uploads/2011/02/Population-Bulletin-2011-66-1-aging-u-s.pdf

[3] Gwira JA Fryar CD Gu Q. Prevalence of Total, diagnosed, and undiagnosed diabetes in adults: United States, august 2021-august 2023. NCHS Data Brief. (2024) 1–11. doi: 10.15620/cdc/165794 PMC12035661 40085919

[4] Caspersen CJ Thomas GD Boseman LA Beckles GL Albright AL. Aging, diabetes, and the public health system in the United States. Am J Public Health. (2012) 102:1482–97. doi: 10.2105/AJPH.2011.300616, 22698044 PMC3464829

[5] Idrees T Castro-Revoredo IA Migdal AL Moreno EM Umpierrez GE. Update on the management of diabetes in long-term care facilities. BMJ Open Diabetes Res Care. (2022) 10:e002705. doi: 10.1136/bmjdrc-2021-002705, 35858714 PMC9305812

[6] Narayan KM Boyle JP Geiss LS Saaddine JB Thompson TJ. Impact of recent increase in incidence on future diabetes burden: U.S., 2005-2050. Diabetes Care. (2006) 29:2114–6. doi: 10.2337/dc06-1136, 16936162

[7] Dao L Choi S Freeby M. Type 2 diabetes mellitus and cognitive function: understanding the connections. Curr Opin Endocrinol Diabetes Obes. (2023) 30:7–13. doi: 10.1097/MED.0000000000000783, 36385094

[8] McCrimmon RJ Ryan CM Frier BM. Diabetes and cognitive dysfunction. Lancet. (2012) 379:2291–9. doi: 10.1016/S0140-6736(12)60360-2, 22683129

[9] Gudala K Bansal D Schifano F Bhansali A. Diabetes mellitus and risk of dementia: a meta-analysis of prospective observational studies. J Diabetes Investig. (2013) 4:640–50. doi: 10.1111/jdi.12087, 24843720 PMC4020261

[10] Zhang Y Chen H Feng Y Liu M Lu Z Hu B . Activation of AMPK by GLP-1R agonists mitigates Alzheimer-related phenotypes in transgenic mice. Nat Aging. (2025) 5:1097–113. doi: 10.1038/s43587-025-00869-3, 40394225

[11] Wong TY Sabanayagam C. Strategies to tackle the global burden of diabetic retinopathy: from epidemiology to artificial intelligence. Ophthalmologica. (2020) 243:9–20. doi: 10.1159/000502387, 31408872

[12] Wang W Lo ACY. Diabetic retinopathy: pathophysiology and treatments. Int J Mol Sci. (2018) 19:1816. doi: 10.3390/ijms19061816, 29925789 PMC6032159

[13] Cheung N Mitchell P Wong TY. Diabetic retinopathy. Lancet. (2010) 376:124–36. doi: 10.1016/s0140-6736(09)62124-3, 20580421

[14] Mohamed Q Gillies MC Wong TY. Management of diabetic retinopathy: a systematic review. JAMA. (2007) 298:902–16. doi: 10.1001/jama.298.8.902, 17712074

[15] Wong TY Liew G Tapp RJ Schmidt MI Wang JJ Mitchell P . Relation between fasting glucose and retinopathy for diagnosis of diabetes: three population-based cross-sectional studies. Lancet. (2008) 371:736–43. doi: 10.1016/S0140-6736(08)60343-8, 18313502 PMC2350208

[16] Wong TY Klein R Islam FM Cotch MF Folsom AR Klein BE . Diabetic retinopathy in a multi-ethnic cohort in the United States. Am J Ophthalmol. (2006) 141:446–55. doi: 10.1016/j.ajo.2005.08.063 16490489 PMC2246042

[17] Li Z Li S Xiao Y Zhong T Yu X Wang L. Nutritional intervention for diabetes mellitus with Alzheimer's disease. Front Nutr. (2022) 9:1046726. doi: 10.3389/fnut.2022.1046726, 36458172 PMC9707640

[18] Otaegui-Arrazola A Amiano P Elbusto A Urdaneta E Martínez-Lage P. Diet, cognition, and Alzheimer's disease: food for thought. Eur J Nutr. (2014) 53:1–23. doi: 10.1007/s00394-013-0561-3, 23892520

[19] Hébert JR Shivappa N Wirth MD Hussey JR Hurley TG. Perspective: the dietary inflammatory index (DII)-lessons learned, improvements made, and future directions. Adv Nutr. (2019) 10:185–95. doi: 10.1093/advances/nmy071, 30615051 PMC6416047

[20] Sudlow C Gallacher J Allen N Beral V Burton P Danesh J . UK biobank: an open access resource for identifying the causes of a wide range of complex diseases of middle and old age. PLoS Med. (2015) 12:e1001779. doi: 10.1371/journal.pmed.1001779, 25826379 PMC4380465

[21] Sun M Wang L Guo Y Yan S Li J Wang X . The association among inflammatory diet, glycohemoglobin, and cognitive function impairment in the elderly: based on the NHANES 2011-2014. J Alzheimer's Dis. (2022) 87:1713–23. doi: 10.3233/JAD-215688, 35491786

[22] Velayudhan L Ryu SH Raczek M Philpot M Lindesay J Critchfield M . Review of brief cognitive tests for patients with suspected dementia. Int Psychogeriatr. (2014) 26:1247–62. doi: 10.1017/S1041610214000416, 24685119 PMC4071993

[23] Liang G Hua R Yang F. Dietary patterns and quality of life among night-shift nurses in tertiary hospitals in Hangzhou: a cross-sectional analysis. Front Public Health. (2025) 13:1638082. doi: 10.3389/fpubh.2025.1638082, 41189976 PMC12580383

[24] Yao F Niu J Zheng Y Shen Y. Pro-inflammatory dietary patterns are associated with dyslipidemia, poor body composition, and sleep quality among healthcare workers: a cross-sectional study. J Hum Nutr Diet. (2025) 38:e70131. doi: 10.1111/jhn.70131, 40988471 PMC12457874

[25] Zhan JJ Hodge RA Dunlop AL Lee MM Bui L Liang D . Dietaryindex: a user-friendly and versatile R package for standardizing dietary pattern analysis in epidemiological and clinical studies. Am J Clin Nutr. (2024) 120:1165–74. doi: 10.1016/j.ajcnut.2024.08.021, 39182618 PMC11600030

[26] Shivappa N Steck SE Hurley TG Hussey JR Hébert JR. Designing and developing a literature-derived, population-based dietary inflammatory index. Public Health Nutr. (2014) 17:1689–96. doi: 10.1017/S1368980013002115, 23941862 PMC3925198

[27] Cicek M Buckley J Pearson-Stuttard J Gregg EW. Characterizing multimorbidity from type 2 diabetes: insights from clustering approaches. Endocrinol Metab Clin N Am. (2021) 50:531–58. doi: 10.1016/j.ecl.2021.05.012, 34399960 PMC8383848

[28] Khunti K Chudasama YV Gregg EW Kamkuemah M Misra S Suls J . Diabetes and multiple long-term conditions: a review of our current Global Health challenge. Diabetes Care. (2023) 46:2092–101. doi: 10.2337/dci23-0035, 38011523 PMC10698221

[29] Kautzky-Willer A Leutner M Harreiter J. Sex differences in type 2 diabetes. Diabetologia. (2023) 66:986–1002. doi: 10.1007/s00125-023-05891-x, 36897358 PMC10163139

[30] Luo Y Li X Yang T Zhou K Wang T Zhang Y . Impact of education level on complications and mortalities in type 2 diabetes: a UK biobank prospective cohort study. BMC Public Health. (2025) 25:3434. doi: 10.1186/s12889-025-24491-5, 41068666 PMC12513123

[31] van Sloten TT Sedaghat S Carnethon MR Launer LJ Stehouwer CDA. Cerebral microvascular complications of type 2 diabetes: stroke, cognitive dysfunction, and depression. Lancet Diabetes Endocrinol. (2020) 8:325–36. doi: 10.1016/S2213-8587(19)30405-X, 32135131 PMC11044807

[32] Fang B Wang Z Nan G. Dietary inflammatory potential and the risk of cognitive impairment: a meta-analysis of prospective cohort studies. J Nutr Health Aging. (2025) 29:100428. doi: 10.1016/j.jnha.2024.100428, 39689376 PMC12180060

[33] Liu S Lu Y Cai X Cong R Li J Jiang H . Glycemic control is related to cognitive dysfunction in elderly people with type 2 diabetes mellitus in a rural Chinese population. Curr Alzheimer Res. (2019) 16:950–62. doi: 10.2174/1567205016666191023110712, 31642779

[34] Wu M Mei F Hu K Feng L Wang Z Gao Q . Diabetic retinopathy and cognitive dysfunction: a systematic review and meta-analysis. Acta Diabetol. (2022) 59:443–59. doi: 10.1007/s00592-021-01829-0, 35112186

[35] Biessels GJ Despa F. Cognitive decline and dementia in diabetes mellitus: mechanisms and clinical implications. Nat Rev Endocrinol. (2018) 14:591–604. doi: 10.1038/s41574-018-0048-7, 30022099 PMC6397437

[36] London A Benhar I Schwartz M. The retina as a window to the brain-from eye research to CNS disorders. Nat Rev Neurol. (2013) 9:44–53. doi: 10.1038/nrneurol.2012.227, 23165340

[37] Ko F Muthy ZA Gallacher J Sudlow C Rees G Yang Q . Association of retinal nerve fiber layer Thinning with current and future cognitive decline: a study using optical coherence tomography. JAMA Neurol. (2018) 75:1198–205. doi: 10.1001/jamaneurol.2018.1578, 29946685 PMC6233846

[38] Forbes JM Cooper ME. Mechanisms of diabetic complications. Physiol Rev. (2013) 93:137–88. doi: 10.1152/physrev.00045.2011, 23303908

[39] Little K Llorián-Salvador M Scullion S Hernández C Simó-Servat O Del Marco A . Common pathways in dementia and diabetic retinopathy: understanding the mechanisms of diabetes-related cognitive decline. Trends Endocrinol Metab. (2022) 33:50–71. doi: 10.1016/j.tem.2021.10.008, 34794851

[40] Kawamura T Umemura T Hotta N. Curious relationship between cognitive impairment and diabetic retinopathy. J Diabetes Investig. (2015) 6:21–3. doi: 10.1111/jdi.12234, 25621129 PMC4296699

[41] Exalto LG Biessels GJ Karter AJ Huang ES Quesenberry CP Jr Whitmer RA. Severe diabetic retinal disease and dementia risk in type 2 diabetes. J Alzheimer's Dis. (2014) 42 Suppl 3:S109–17. doi: 10.3233/JAD-132570 24625797 PMC4373321

[42] Cavicchia PP Steck SE Hurley TG Hussey JR Ma Y Ockene IS . A new dietary inflammatory index predicts interval changes in serum high-sensitivity C-reactive protein. J Nutr. (2009) 139:2365–72. doi: 10.3945/jn.109.114025, 19864399 PMC2777480

[43] Mayr HL Itsiopoulos C Tierney AC Ruiz-Canela M Hebert JR Shivappa N . Improvement in dietary inflammatory index score after 6-month dietary intervention is associated with reduction in interleukin-6 in patients with coronary heart disease: the AUSMED heart trial. Nutr Res. (2018) 55:108–21. doi: 10.1016/j.nutres.2018.04.007, 29807669

[44] Kanauchi M Shibata M Iwamura M. A novel dietary inflammatory index reflecting for inflammatory ageing: technical note. Ann Med Surg (Lond). (2019) 47:44–6. doi: 10.1016/j.amsu.2019.09.012, 31641503 PMC6796497

[45] Almeida-de-Souza J Santos R Barros R Abreu S Moreira C Lopes L . Dietary inflammatory index and inflammatory biomarkers in adolescents from LabMed physical activity study. Eur J Clin Nutr. (2018) 72:710–9. doi: 10.1038/s41430-017-0013-x, 29277838

[46] Corley J Shivappa N Hébert JR Starr JM Deary IJ. Associations between dietary inflammatory index scores and inflammatory biomarkers among older adults in the Lothian birth cohort 1936 study. J Nutr Health Aging. (2019) 23:628–36. doi: 10.1007/s12603-019-1221-y, 31367727 PMC6675764

[47] Bonnefont-Rousselot D. Glucose and reactive oxygen species. Curr Opin Clin Nutr Metab Care. (2002) 5:561–8. doi: 10.1097/00075197-200209000-00016, 12172481

[48] Jackson GR Werrbach-Perez K Pan Z Sampath D Perez-Polo JR. Neurotrophin regulation of energy homeostasis in the central nervous system. Dev Neurosci. (1994) 16:285–90. doi: 10.1159/000112121, 7768207

[49] Mittal M Siddiqui MR Tran K Reddy SP Malik AB. Reactive oxygen species in inflammation and tissue injury. Antioxid Redox Signal. (2014) 20:1126–67. doi: 10.1089/ars.2012.5149, 23991888 PMC3929010

[50] Xu H Chen M Forrester JV. Para-inflammation in the aging retina. Prog Retin Eye Res. (2009) 28:348–68. doi: 10.1016/j.preteyeres.2009.06.001, 19560552

[51] Zheng Y Ley SH Hu FB. Global aetiology and epidemiology of type 2 diabetes mellitus and its complications. Nat Rev Endocrinol. (2018) 14:88–98. doi: 10.1038/nrendo.2017.151 29219149

[52] Liu RH. Health benefits of fruit and vegetables are from additive and synergistic combinations of phytochemicals. Am J Clin Nutr. (2003) 78:517s–20s. doi: 10.1093/ajcn/78.3.517S, 12936943

[53] Eberhardt MV Lee CY Liu RH. Antioxidant activity of fresh apples. Nature. (2000) 405:903–4. doi: 10.1038/35016151, 10879522

[54] Slavin J. Whole grains and human health. Nutr Res Rev. (2004) 17:99–110. doi: 10.1079/nrr200374, 19079919

