# Supplementary material for: Associations of diabetes mellitus with/without diabetic retinopathy and cognitive outcomes in older adults: the potential role of the dietary inflammatory index in a multi-dataset observational study
Source: Front Nutr. 2026 May 12;13:1775880. doi: 10.3389/fnut.2026.1775880 (PMC13201100; doi:10.3389/fnut.2026.1775880)
Supplement: Supplementary file 1 [file Supplementary_file_1.docx]

**Supplementary Methods**

**Cognitive assessment**

The CERAD evaluates the immediate and delayed learning abilities for new verbal information, including three consecutive learning trials and one delayed recall trial (1). In the learning trials, participants were asked to read 10 unrelated words aloud, one at a time. After the presentation of words, they were immediately asked to recall as many as possible. The sequence of the 10 words was altered in each of the three learning trials. The maximum score for each trial was 10. The delayed word recall trial was conducted after the AFT and DSST. Each trial had a score range from 0 to 10, and the sum of the three immediate trials and one delayed trial constituted the total CERAD score. AFT assesses categorical verbal fluency as a component of executive function (2). Participants were asked to name as many animals as they could within 1 minute. The score was the total number of correct answers. The DSST measures processing speed, sustained attention, and working memory (3). This exercise was conducted using a paper form with a key at the top containing nine numbers and corresponding symbols. Participants have 2 min to match the correct symbols with the numbers in 133 boxes next to the numbers, and the score is the total number of correct matches. A practice trial is conducted before the formal test begins.

There are currently no official education-adjusted diagnostic cutoff values or standardized composite impairment algorithms for the NHANES cognitive battery including the DSST, CERAD, and AFT. Following prior NHANES-based studies (4-6) and the CDC/NCHS reporting framework, we used the survey-weighted lowest 25th percentile of test scores as the cutoff to define low cognitive performance. As age was the main risk factor for cognitive performance and the subjects included in this study were 60–80 years old, participants were divided into two age groups (60–69 years and 70–80 years). The lowest 25th percentile within each group was used as the cutoff value to define low cognitive performance. The cutoff values for low cognitive performance for the two age groups in the CERAD test were 22 and 19, respectively, those in the AFT were 14 and 11, respectively, and those in the DSST were 39 and 28, respectively.

**Dietary Inflammatory Index (DII)**

The DII was developed to compare the dietary inflammatory potential of different populations through a literature review by Shivappa (7). By screening the effects of different dietary parameters on six inflammatory markers (IL-1β, IL-4, IL-6, IL-10, TNF-α, and CRP), 45 dietary parameters were determined to establish the DII algorithm. A score of +1 is given if these parameters are proinflammatory (increased IL-1β, IL-6, TNF-α, or CRP, or decreased IL-4 or IL-10); a score of –1 is given if the effect is anti-inflammatory (reduced IL-1β, IL-6, TNF, or CRP, or increased IL-4 or IL-10); and a score of 0 is given if there are no significant changes in inflammatory markers.

The number and type of nutrient parameters available for DII calculation differed across datasets because the dietary instruments and food composition databases were not identical. Therefore, we calculated the dataset-specific DII using only the eligible parameters available in each dataset, and unavailable components were not included. In NHANES, the DII was calculated using 28 diet or nutrient parameters, which included energy, carbohydrate, dietary fiber, protein, total fat, saturated fatty acids, monounsaturated fatty acids, polyunsaturated fatty acids, n-3 fatty acids, n-6 fatty acids, cholesterol, Vitamin A/D/E/B6/B12/C, β-carotene, thiamin, riboflavin, nicotinic acids, folic acids, iron, magnesium, zinc, selenium, caffeine, and alcohol. According to the UK Nutrient Databank food composition tables, nutrient intake was estimated by multiplying the portions consumed by the specified quantity and nutrient composition. Ultimately, 28 eligible nutritional parameters were available in the UKB dataset to calculate the DII, which include energy, alcohol, carbohydrate, protein, fat, saturated fatty acids, monounsaturated fatty acids, trans fatty acids, n-3 fatty acids, n6 fatty acids, Englyst fiber, cholesterol, β-carotene, Vitamin A retinol equivalents, Vitamin B6/B12/C/D/E, niacin equivalent, selenium, thiamin, riboflavin, folate, iron, magnesium, zinc, tea. In the Wenzhou dataset, dietary intake was assessed using the FFQ-25, which captured habitual dietary intake during the previous 6 months. The questionnaire consists of 50 items covering the consumption of 25 food categories, including rice, porridge, flour-based foods, sweets, fried foods, processed foods, whole grains, tubers, milk, eggs, red meat, poultry, processed meats, freshwater and marine seafood, soy products, nuts, dark and light vegetables, mushrooms, fruits, sugary drinks, beer, yellow wine, and liquor. According to the study of Gao et al. (8), food consumption frequency was converted into frequency weights as follows: never (f1 = 0); less than once a month (f1 = 0.03); 1-3 times a month (f1 = 0.07); 1-2 times a week (f1 = 0.22); 3-4 times a week (f1 = 0.50); 5-6 times a week (f1 = 0.79); once a day (f1 = 1); twice a day (f1 = 2); more than three times a day (f1 = 3). Food intake amounts were assigned intake weights as follows: up to 50g (f2 = 0.50); 100g (f2 = 0.75); 150g (f2 = 1.00); 200g (f2 = 1.50); and 250g or more (f2 = 2.00). In total, 16 nutrient-derived parameters were selected for DII calculation in the Wenzhou dataset. To improve cross-dataset comparability, these parameters were selected from the China Food Composition Standard Edition (6th edition) on the basis of their overlap with the DII-related nutrient components available in NHANES and the UKB dataset, including energy, protein, fat, carbohydrates, insoluble dietary fiber, cholesterol, Vitamin A, vitamin C, β-carotene, thiamine, riboflavin, niacin acids, magnesium, iron, zinc, and selenium. Nutrient intake for each food was calculated by multiplying its frequency weight, intake weight, and nutrient content, and these values were then used to derive the DII.

**Exploratory segmented/breakpoint analysis**

Because the primary restricted cubic spline curve suggested that the association between DII and CFI was relatively flat at lower DII values and became more clearly positive at higher DII levels, we prespecified one candidate breakpoint interval rather than testing multiple ranges. The interval of 2.4-3.2 was chosen to cover the plausible slope-change region in Figure 3A while avoiding the sparse tails of the DII distribution. This strategy was used to reduce data-driven model selection and to limit model flexibility.

Within this prespecified interval, the initial breakpoint value was set at 2.8, corresponding to the midpoint of the candidate range. Two exploratory segmented analyses were then conducted. First, a fixed-breakpoint survey-weighted segmented logistic regression model was fitted using the prespecified breakpoint of 2.8. Second, a grid-search procedure was performed across the same interval (2.4-3.2) in 0.05-unit increments, and the breakpoint with the best survey-based Akaike information criterion (AIC) was selected. In both segmented models, odds ratios (ORs) below and above the breakpoint were calculated from the corresponding regression coefficients. The significance of the slope change was assessed using a Wald test for the hinge term. Segmented and linear survey-weighted logistic regression models were compared using design-based Wald tests and survey-based AIC. These analyses were considered exploratory and were used to assess whether a threshold-like change in slope could be estimated, rather than to define a definitive clinical cutoff.

| Table S1. ICD-10 codes used to ascertain all-cause dementia cases and DM. | | |
| --- | --- | --- |
| Clinical diagnosis | ICD CODE (Data-Field 41270) | Diagnosis description |
| All-cause dementia | A81.0 | Sporadic Creutzfeldt-Jakob disease |
|  | F00 | Dementia in Alzheimer's disease |
|  | F00.0 | Dementia in Alzheimer's disease with early onset |
|  | F00.1 | Dementia in Alzheimer's disease with late onset |
|  | F00.2 | Dementia in Alzheimer's disease, atypical or mixed type |
|  | F00.9 | Dementia in Alzheimer's disease, unspecified |
|  | F01 | Vascular dementia |
|  | F01.0 | Vascular dementia of acute onset |
|  | F01.1 | Multi-infarct dementia |
|  | F01.2 | Subcortical vascular dementia |
|  | F01.3 | Mixed cortical and sub-cortical vascular dementia |
|  | F01.8 | Other vascular dementia |
|  | F01.9 | Vascular dementia, unspecified |
|  | F02 | Dementia in other diseases classified elsewhere |
|  | F02.0 | Dementia in Picks disease |
|  | F02.1 | Dementia in Creutzfeldt-Jacob disease |
|  | F02.2 | Dementia in Huntington’s disease |
|  | F02.3 | Dementia in Parkinson’s disease |
|  | F02.4 | Dementia in HIV disease |
|  | F02.8 | Dementia in other specified diseases classified elsewhere |
|  | F03 | Unspecified dementia |
|  | F05.1 | Delirium superimposed on dementia |
|  | F10.6 | Mental and behavioural disorders due to use of alcohol - amnesic syndrome |
|  | G30 | Alzheimer’s disease |
|  | G30.0 | Alzheimer’s disease with early onset |
|  | G30.1 | Alzheimer’s disease with late onset |
|  | G30.8 | Other Alzheimer's disease |
|  | G30.9 | Alzheimer's disease unspecified |
|  | G31.0 | Circumscribed brain atrophy |
|  | G31.1 | Senile degeneration of brain |
|  | G31.8 | Other specified degenerative diseases of nervous system |
|  | I67.3 | Binswanger's disease |
| Diabetes Mellitus | E10 | Insulin-dependent diabetes mellitus |
|  | E11 | Non-insulin-dependent diabetes mellitus |
|  | E12 | Malnutrition-related diabetes mellitus |
|  | E13 | Other specified diabetes mellitus |
|  | E14 | Unspecified diabetes mellitus |

| Table S2. Definitions and assessments of covariates and outcome ascertainment variables. | | | |
| --- | --- | --- | --- |
| Covariates | Definition | Assessment | UK biobank Data-Field ID |
| Age (years) | Age in years. | The age of the participant on the day they attended an Initial Assessment Centre | Data-Field 21022 |
| Sex | Men, Women. | NHS derived and/or self-reported Gender. | Data-Field 31 |
| Race/ethnicity | White (British, Irish, White, Any other white background), Non-White | Touchscreen questionnaire: “What is your ethnic group?”. | Data-Field 21000 |
| Education years | Years of education. | ACE Touchscreen questionnaire: “Which of the following qualifications do you have?”.  Education levels were further converted to education years based on the following previously described rules (9): 20 years for a college or university degree; 18 years for professional qualifications, such as nursing or teaching; 13 years for A levels/AS levels, CSEs, NVQ, HND, or HNC; 10 years for O levels/GCSEs or equivalents; and 7 years for no aforementioned qualifications. | Data-Field 6138 |
| BMI | Body Mass Index. Units of measurement are Kg/m^2^. | BMI value here is constructed from height and weight measured during the initial Assessment Centre visit. Value is not present if either of these readings were omitted.  According to the World Health Organization (WHO) classification, Body Mass Index (BMI) is categorized into four groups: underweight (BMI < 18.5 kg/m²), normal range (BMI 18.5–24.9 kg/m²), overweight (BMI 25.0–29.9 kg/m²), and obesity (BMI ≥ 30.0 kg/m²). | Data-Field 21001 |
| Smoking status | Yes, No. | ACE touchscreen question "Do you smoke tobacco now?" | Data-Field 1239 |
| Drinking status | Yes, No. | ACE Touchscreen questionnaire: “About how often do you drink alcohol?”. | Data-Field 1558 |
| DM | Yes, No. | Diseases ascertained by ICD-10 codes: Insulin-dependent diabetes mellitus; Non-insulin-dependent diabetes mellitus; Malnutrition-related diabetes mellitus; Other specified diabetes mellitus; Unspecified diabetes mellitus (ICD code E10, E11, E12, E13, E14). | Data-Field 41270 |
| Glycated haemoglobin (HbA1c) | Continuous, mmol/mol | Measured by HPLC analysis on a Bio-Rad VARIANT II Turbo | Data-Field 30750 |
| Diagnoses - ICD10 | Categorical (multiple) | a summary of the distinct diagnosis codes a participant has had recorded across all their hospital inpatient records in either the primary or secondary position | Data-Field 41270 |
| Date of first in-patient diagnosis - ICD10 | Date | the date each ICD-10 diagnosis code was first recorded in either the primary or secondary position in the participant's hospital inpatient records | Data-Field 41280 |

**Supplementary results**

**Firth penalized conditional logistic regression analysis in the Wenzhou dataset**

To further assess the potential impact of overfitting in the Wenzhou dataset, we performed Firth penalized conditional logistic regression as a sensitivity analysis and compared the estimates with those from the standard conditional logistic regression. The results were materially similar across methods. For DR, the ORs from the standard conditional logistic regression were 6.998 (95% CI: 3.535-13.854) in Model 2 and 7.555 (95% CI: 3.706-15.401) in Model 3 (Table S5), whereas the corresponding estimates from the Firth penalized models were 7.715 (95% CI: 3.737-15.927) and 7.666 (95% CI: 3.692-15.920), respectively (Table S6). For DII, the ORs were 10.241 (95% CI: 3.475-30.186) and 12.642 (95% CI: 3.987-40.080) in the standard models, compared with 11.338 (95% CI: 3.506-36.665) and 12.699 (95% CI: 3.775-42.725) in the Firth models. HbA1c and HOMA-IR also showed similar estimates across methods and remained non-significant after multivariable adjustment. Overall, these findings indicate that the results were not materially altered by penalization, suggesting that the standard conditional logistic regression estimates were reasonably stable in the revised matched sample.

**Exploratory segmented/breakpoint analysis**

In the survey-weighted logistic regression model (Table S11), DII was positively associated with CFI (OR = 1.34, 95% CI: 1.23-1.47). In the exploratory survey-weighted segmented logistic regression model with a prespecified breakpoint at 2.8, the association was weaker below the breakpoint (OR = 1.25, 95% CI: 1.13-1.37) and stronger above the breakpoint (OR = 2.12, 95% CI: 1.51-2.97), with a significant change in slope (P = 0.010). The segmented model also showed better fit than the linear model (AIC 2774.0 vs 2780.8) (Table S11).

In the grid-search analysis restricted to the same prespecified interval of 2.4-3.2, the best-fitting breakpoint was 2.4. Below this value, the OR was 1.23 (95% CI: 1.11-1.35), whereas above it the OR was 1.83 (95% CI: 1.42-2.34), again suggesting a steeper positive association at higher DII levels (Table S11). However, because the best-fitting breakpoint was located at the boundary of the candidate interval, we do not interpret this result as evidence of a clear or robust threshold. Overall, these exploratory segmented analyses were broadly consistent with the spline findings and supported a nonlinear positive association, but they did not identify a single clear threshold.


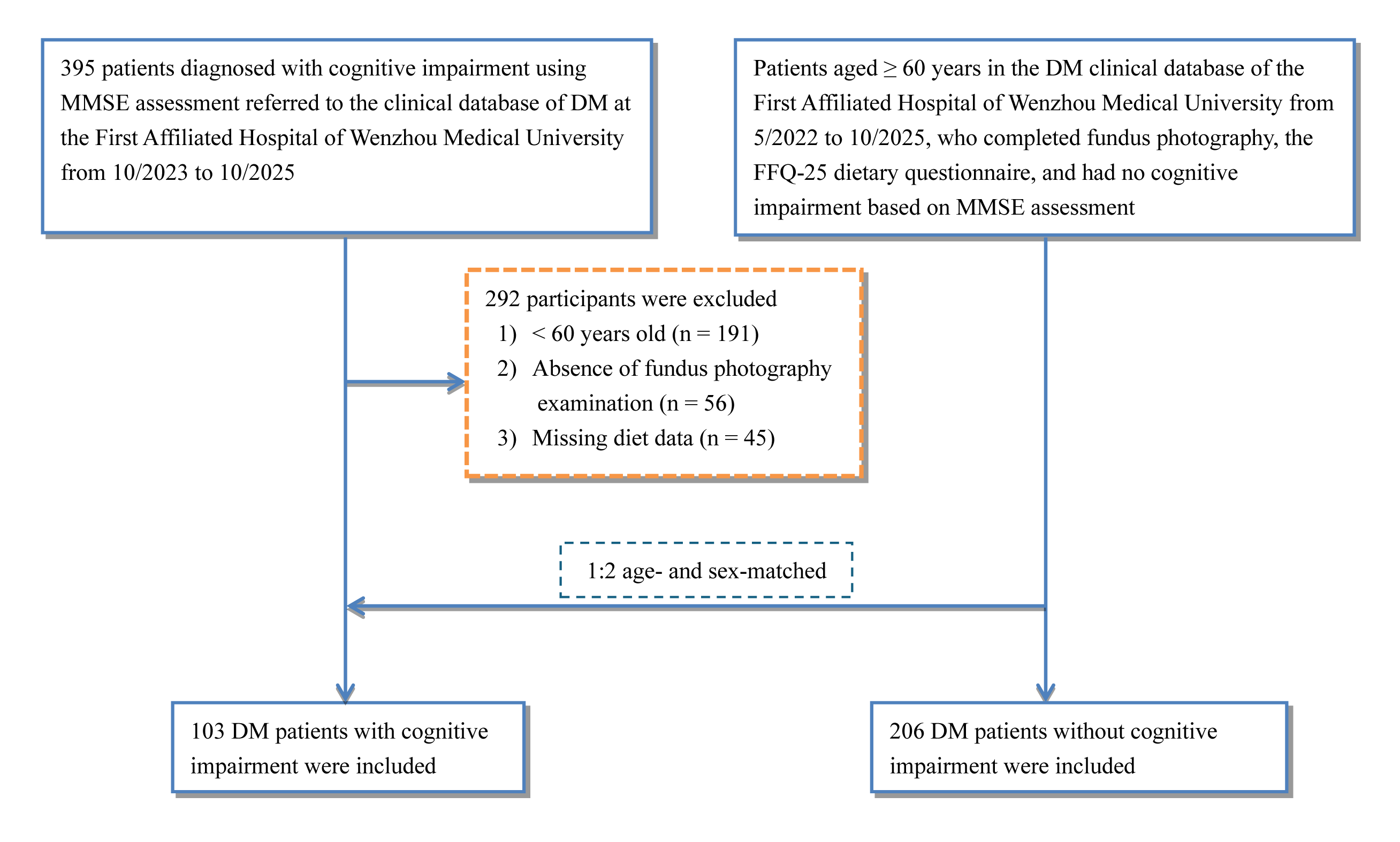


**Figure S1.** Flow diagram of the Wenzhou dataset. DM, diabetes mellitus; MMSE, Mini-Mental State Examination.

**
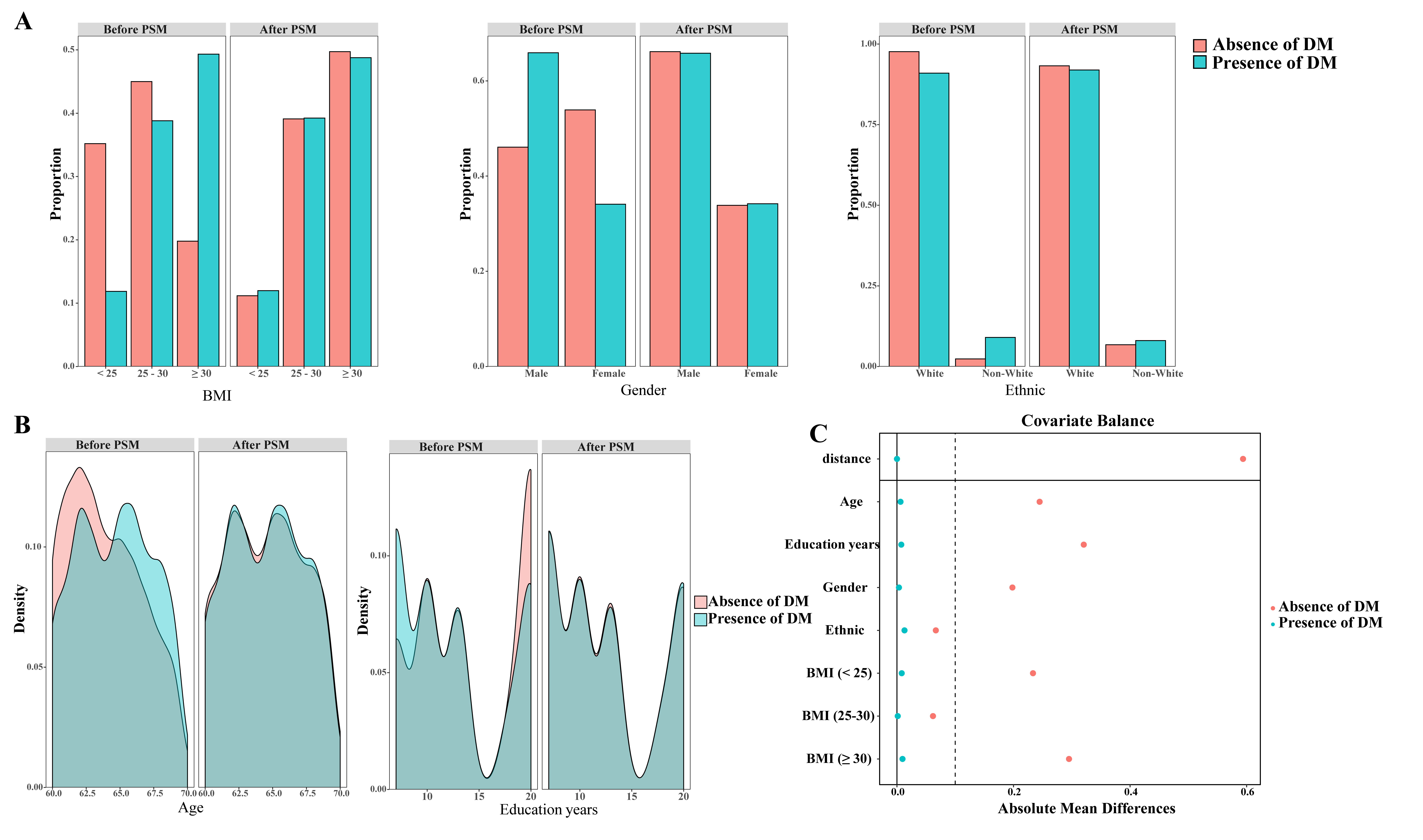
**

**Figure S2.** Covariate balance diagnostics before and after propensity score matching in the UKB dataset. (A) Distribution of categorical covariates, including BMI group, sex, and ethnicity, in participants with and without DM before and after PSM. (B) Density plots showing the distributions of continuous covariates, including age and educational years, before and after PSM. (C) Love plot displaying the absolute standardized mean differences of baseline covariates before and after PSM. The marked reduction in standardized mean differences after matching indicates improved covariate balance between participants with and without DM.

**

**

**Figure S3.** Sensitivity analyses of the nonlinear association between the DII and CFI using 4-knot and 6-knot restricted cubic spline models in NHANES participants. (A) 4-knot model in all participants, with knots placed at the 5th, 35th, 65th, and 95th percentiles of the DII distribution. (B) 6-knot model in all participants, with knots placed at the 5th, 23rd, 41st, 59th, 77th, and 95th percentiles of the DII distribution. (C) 4-knot model among participants with diabetes and available DR data. (D) 6-knot model among participants with diabetes and available DR data. Solid lines represent adjusted odds ratios (ORs), and shaded areas indicate 95% confidence intervals (CIs). The vertical dashed line indicates the reference value, defined as the median DII of each analytic sample, and the horizontal dashed line indicates an OR of 1.

| Table S3. Comparison of baseline characteristics between included and excluded participants in the NHANES dataset | | | |
| --- | --- | --- | --- |
| Variables | Included participants  Mean (SE) or % (95% CI) | Excluded participants  Mean (SE) or % (95% CI) | P-value |
| Age (years) | 69.2 (0.2) | 69.8 (0.3) | 0.165 |
| Gender (male) | 45.7 (43.9 ,47.6) | 46.2 (40.6 ,51.8) | 0.877 |
| Race/ethnicity |  |  | <0.001 |
| Mexican American | 3.5 (2.1 ,5.8) | 5.3 (3.4 ,8.1) |  |
| Other Hispanic | 3.8 (2.5 ,5.7) | 4.7 (3.1 ,7.1) |  |
| Non-Hispanic White | 79.3 (74.8 ,83.1) | 65.5 (57.9 ,72.5) |  |
| Non-Hispanic Black | 8.2 (6.0 ,10.9) | 14.6 (10.1 ,20.5) |  |
| Non-Hispanic Asian | 3.7 (2.8 ,4.9) | 6.3 (4.1 ,9.6) |  |
| Other Race | 1.5 (0.9 ,2.5) | 3.6 (1.3 ,9.1) |  |
| Education level |  |  | 0.164 |
| Below High school | 16.3 (13.3 ,19.9) | 20.5 (16.4 ,25.4) |  |
| High school | 22.2 (19.6 ,25.1) | 23.0 (18.0 ,28.8) |  |
| Above High school | 61.5 (57.9 ,64.9) | 56.5 (50.1 ,62.6) |  |
| HbA1c, % | 5.6 (0.1) | 6.1 (0.1) | 0.008 |
| BMI, kg/m² | 29.0 (0.2) | 29.1 (0.5) | 0.850 |
| Smoking status | 50.3 (47.2 ,53.3) | 48.8 (41.5 ,56.2) | 0.687 |
| Drinking status | 72.3 (69.2 ,75.2) | 63.2 (57.1 ,68.9) | <0.001 |
| DM | 20.6 (19.0 ,22.3) | 21.4 (17.8 ,25.6) | 0.666 |
| DR | 16.0 (13.8 ,18.5) | 21.9 (13.0 ,34.5) | 0.165 |
| NOTE: Continuous variables are presented as weighted means (weighted standard errors) and categorical variables as weighted percentages (95% CI). BMI: Body Mass Index; CI, Confidence Interval; DM: Diabetes Mellitus; DR: diabetic retinopathy; | | | |

| Table S4. Multivariable robust Cox proportional hazards analysis of factors associated with all-cause dementia in UKB dataset (N=2201) | | | | | | |
| --- | --- | --- | --- | --- | --- | --- |
| Variables | Model 1^a^ |  | Model 2^b^ |  | Model 3^c^ |  |
|  | HR (95% CI) | P-value | HR (95% CI) | P-value | HR (95% CI) | P-value |
| DM | 2.684 (1.628-4.423) | <0.001 | 2.722 (1.65-4.489) | <0.001 | 2.711 (1.641-4.478) | <0.001 |
| DII | 1.211 (1.088-1.349) | <0.001 | 1.202 (1.081-1.336) | <0.001 | 1.201 (1.072-1.346) | 0.002 |
| HbA1c | 1.025 (1.011-1.04) | <0.001 | 1.027 (1.012-1.042) | <0.001 | 1.027 (1.012-1.042) | <0.001 |
| NOTE: CI, Confidence Interval; DII: Dietary Inflammatory Index; DM: Diabetes Mellitus.  ^a^ Model 1: conditioned on matched sets only.  ^b^ Model 2: adjusted for age, sex, educational years, BMI.  ^c^ Model 3: adjusted for age, sex, educational years, BMI, race, smoking status and drinking status. | | | | | | |

| Table S5. Conditional logistic regression analysis for the association between factors and Cognitive impairment in Wenzhou dataset | | | | | | |
| --- | --- | --- | --- | --- | --- | --- |
| Variables | Model 1^a^ |  | Model 2^b^ |  | Model 3^c^ |  |
|  | OR (95% CI) | P-value | OR (95% CI) | P-value | OR (95% CI) | P-value |
| DR | 5.592 (3.011-10.386) | <0.001 | 6.998 (3.535-13.854) | <0.001 | 7.555 (3.706-15.401) | <0.001 |
| DII | 8.947 (3.204-24.983) | <0.001 | 10.241 (3.475-30.186) | <0.001 | 12.642 (3.987-40.08) | <0.001 |
| HbA1c | 1.117 (1.003-1.245) | 0.043 | 1.080 (0.966-1.207) | 0.175 | 1.048 (0.934-1.177) | 0.425 |
| HOMA-IR | 0.946 (0.846-1.058) | 0.333 | 0.973 (0.865-1.095) | 0.649 | 0.953 (0.842-1.078) | 0.440 |
| NOTE: CI, Confidence Interval; DII: Dietary Inflammatory Index; DR: Diabetic Retinopathy. HOMA-IR, Homeostasis model assessment of insulin resistance.  ^a^ Model 1: conditioned on matched sets only.  ^b^ Model 2: adjusted for educational years, BMI.  ^c^ Model 3: adjusted for educational years, BMI, smoking status, drinking status and diabetes duration. | | | | | | |

| Table S6. Firth penalized conditional logistic regression for the association between factors and Cognitive impairment in Wenzhou dataset | | | | | | |
| --- | --- | --- | --- | --- | --- | --- |
| Variables | Model 1^a^ |  | Model 2^b^ |  | Model 3^c^ |  |
|  | OR (95% CI) | P-value | OR (95% CI) | P-value | OR (95% CI) | P-value |
| DR | 6.464 (3.259-12.820) | <0.001 | 7.715 (3.737-15.927) | <0.001 | 7.666 (3.692-15.920) | <0.001 |
| DII | 10.105 (3.287-31.061) | <0.001 | 11.338 (3.506-36.665) | <0.001 | 12.699 (3.775-42.725) | <0.001 |
| HbA1c | 1.131 (0.994-1.287) | 0.063 | 1.090 (0.955-1.245) | 0.201 | 1.062 (0.926-1.217) | 0.392 |
| HOMA-IR | 0.943 (0.828-1.074) | 0.376 | 0.974 (0.850-1.116) | 0.706 | 0.955 (0.832-1.098) | 0.519 |
| NOTE: CI, Confidence Interval; DII: Dietary Inflammatory Index; DR: Diabetic Retinopathy. HOMA-IR, Homeostasis model assessment of insulin resistance.  ^a^ Model 1: unadjusted model.  ^b^ Model 2: adjusted for educational years, BMI.  ^c^ Model 3: adjusted for educational years, BMI, smoking status, drinking status and diabetes duration. | | | | | | |

| Table S7. Exploratory mediation analysis of DII and HbA1c in the association between DM and cognitive performance among NHANES participants (N=2524) | | | | | | |
| --- | --- | --- | --- | --- | --- | --- |
| Outcomes | Exposure | Mediators | Indirect effects (95%CI) | Direct effects (95%CI) | Mediated proportion (%) | P-value |
| CFI | DM | DII | 0.012 (0.006-0.019) ^**^ | 0.067 (0.024-0.108) ^**^ | 15.0% | 0.002 |
|  | DM | HbA1c | 0.019 (-0.009-0.05) | 0.060 (0.012-0.113) ^*^ | 24.1% | 0.210 |
| CERAD | DM | DII | 0.009 (0.004-0.015) ^**^ | 0.048 (0.010-0.088) ^*^ | 15.4% | 0.008 |
|  | DM | HbA1c | 0.014 (-0.011-0.041) | 0.042 (-0.003-0.088) | 25.3% | 0.298 |
| AFT | DM | DII | 0.010 (0.005-0.016) ^**^ | 0.044 (0.008-0.079) ^*^ | 18.7% | 0.008 |
|  | DM | HbA1c | 0.003 (-0.022-0.026) | 0.051 (0.008-0.094) ^*^ | 4.7% | 0.866 |
| DSST | DM | DII | 0.008 (0.003-0.013) ^**^ | 0.057 (0.019-0.094) ^**^ | 11.9% | 0.002 |
|  | DM | HbA1c | 0.027 (0.005-0.049) ^*^ | 0.036 (-0.008-0.081) | 43.1% | 0.022 |
| NOTE: AFT: Animal Fluency Test; CERAD: Consortium to Establish a Registry for Alzheimer’s Disease; CFI: Cognitive Function Impairment; CI, Confidence Interval; DII: Dietary Inflammatory Index; DM: Diabetes Mellitus; DSST: Digit Symbol Substitution Test;  This Model was adjusted for age, sex, race, educational level, BMI, smoking status, drinking status. *p < 0.05, **p < 0.01 and ***p < 0.001. | | | | | | |

| Table S8. Exploratory mediation analysis of DII and HbA1c in the association between DR and cognitive performance in DM participants with/without DR in NHANES dataset (N=628) | | | | | | |
| --- | --- | --- | --- | --- | --- | --- |
| Outcomes | Exposure | Mediators | Indirect effects (95%CI) | Direct effects (95%CI) | Mediated proportion (%) | P-value |
| CFI | DR | DII | 0.010 (0.000-0.022) ^*^ | 0.113 (0.027-0.202) ^*^ | 8.0% | 0.048 |
|  | DR | HbA1c | 0.002 (-0.006-0.012) | 0.120 (0.033-0.207) ^*^ | 1.8% | 0.562 |
| CERAD | DR | DII | 0.007 (0.000-0.020) | 0.096 (0.006-0.185) ^*^ | 7.2% | 0.100 |
|  | DR | HbA1c | 0.003 (-0.004-0.015) | 0.101 (0.011-0.189) ^*^ | 2.9% | 0.434 |
| AFT | DR | DII | 0.017 (0.001-0.036) ^*^ | 0.018 (-0.072-0.098) | 48.7% | 0.480 |
|  | DR | HbA1c | -0.002 (-0.012-0.006) | 0.039 (-0.054-0.122) | -4.4% | 0.810 |
| DSST | DR | DII | 0.010 (0.001-0.023) ^*^ | 0.041 (-0.043-0.121) | 20.5% | 0.274 |
|  | DR | HbA1c | 0.006 (-0.001-0.019) | 0.044 (-0.039-0.123) | 11.8% | 0.348 |
| NOTE: AFT: Animal Fluency Test; CERAD: Consortium to Establish a Registry for Alzheimer’s Disease; CFI: Cognitive Function Impairment; CI, Confidence Interval; DII: Dietary Inflammatory Index; DR: diabetic retinopathy; DSST: Digit Symbol Substitution Test;  This Model was adjusted for age, sex, race, educational level, BMI, smoking status, drinking status. *p < 0.05, **p < 0.01 and ***p < 0.001. | | | | | | |

| Table S9. Exploratory mediation analysis of DII and HbA1c in the association between DM and all-cause dementia in the UKB dataset (N=2201) | | | | |
| --- | --- | --- | --- | --- |
| Mediators | Indirect effects (95%CI) | Direct effects (95%CI) | Mediated proportion (%) | P-value |
| DII | 0.001 (0.000-0.003) ^*^ | 0.025 (0.010-0.043) ^***^ | 4.1% | 0.028 |
| HbA1c | 0.000 (-0.014-0.010) | 0.027 (0.008-0.049) ^**^ | 0.6% | 0.980 |
| NOTE: CI, Confidence Interval; DII: Dietary Inflammatory Index; DM: Diabetes Mellitus. This model was adjusted for age, sex, race, educational years, BMI, smoking status and drinking status. *p < 0.05, **p < 0.01, ***p < 0.001. | | | | |

| Table S10. Exploratory mediation analysis of DII, HbA1c, and HOMA-IR in the association between DR and cognitive impairment in the Wenzhou dataset | | | | |
| --- | --- | --- | --- | --- |
| Mediators | Indirect effects (95%CI) | Direct effects (95%CI) | Mediated proportion (%) | P-value |
| DII | 0.496 (0.162, 1.042) ^***^ | 5.549 (2.683, 11.478) ^***^ | 13.6% | <0.001 |
| HbA1c, % | 0.017 (-0.055, 0.102) | 7.255 (3.606, 14.598) ^***^ | 0.1% | 0.635 |
| HOMA-IR | 0.011 (-0.061, 0.092) | 7.242 (3.602, 14.560) ^***^ | 0.2% | 0.745 |
| NOTE: CI, Confidence Interval; DII: Dietary Inflammatory Index; DR: Diabetic Retinopathy. Given the matched case-control design, direct and total effects were estimated using conditional logistic regression models stratified by matched sets and are presented as odds ratios. Indirect effects were estimated using the product-of-coefficients approach, as mediation through a continuous mediator is not directly expressed on the odds ratio scale in conditional logistic regression. The mediated proportion was calculated based on the reduction of the log odds ratio after inclusion of the mediator and should be interpreted as an exploratory measure rather than an exact causal decomposition of the total effect. This model was adjusted for educational years, BMI, smoking status, drinking status and diabetes duration. *p < 0.05, **p < 0.01, ***p < 0.001. | | | | |

| Table S11. Survey-weighted logistic regression analyses for the association between DII and CFI among NHANES participants (N=2524) | | | | | |
| --- | --- | --- | --- | --- | --- |
| Analysis | Breakpoint specification | Estimated breakpoint | OR below breakpoint (95% CI) | OR above breakpoint (95% CI) | P for slope change |
| Survey-weighted logistic regression | None | NA | 1.34 (1.23, 1.47) | NA | NA |
| Survey-weighted segmented logistic regression | Fixed breakpoint at 2.8 | 2.8 | 1.25 (1.13, 1.37) | 2.12 (1.51, 2.97) | 0.010 |
| Survey-weighted grid-search segmented logistic regression | Breakpoint searched within prespecified interval 2.4–3.2 | 2.4 | 1.23 (1.11, 1.35) | 1.83 (1.42, 2.34) | 0.011 |
| NOTE: Models were fitted using the NHANES complex sampling design with survey weights. All models were adjusted for age, sex, race, educational level, BMI, smoking status and drinking status. The fixed-breakpoint model used a prespecified breakpoint of 2.8. The grid-search model restricted candidate breakpoints to the interval 2.4-3.2 in 0.05-unit increments and selected the breakpoint with the best survey-based AIC. CFI: Cognitive Function Impairment; DII: Dietary Inflammatory Index. | | | | | |

**References**

1. Morris JC, Mohs RC, Rogers H, Fillenbaum G, Heyman A. Consortium to establish a registry for Alzheimer's disease (CERAD) clinical and neuropsychological assessment of Alzheimer's disease. Psychopharmacol Bull. 1988;24(4):641-52.

2. Morris JC, Heyman A, Mohs RC, Hughes JP, van Belle G, Fillenbaum G, et al. The Consortium to Establish a Registry for Alzheimer's Disease (CERAD). Part I. Clinical and neuropsychological assessment of Alzheimer's disease. Neurology. 1989;39(9):1159-65.

3. Wechsler D. Wechsler Adult Intelligence Scale-Fourth Edition (WAIS-IV). 2008.

4. Gong Z, Song W, Gu M, Zhou X, Tian C. Association between serum iron concentrations and cognitive impairment in older adults aged 60 years and older: A dose-response analysis of National Health and Nutrition Examination Survey. PLoS One. 2021;16(8):e0255595.

5. Wang X, Xiao P, Wang R, Luo C, Zhang Z, Yu S, et al. Relationships between urinary metals concentrations and cognitive performance among U.S. older people in NHANES 2011-2014. Front Public Health. 2022;10:985127.

6. Li S, Sun W, Zhang D. Association of Zinc, Iron, Copper, and Selenium Intakes with Low Cognitive Performance in Older Adults: A Cross-Sectional Study from National Health and Nutrition Examination Survey (NHANES). J Alzheimers Dis. 2019;72(4):1145-57.

7. Shivappa N, Steck SE, Hurley TG, Hussey JR, Hébert JR. Designing and developing a literature-derived, population-based dietary inflammatory index. Public health nutrition. 2014;17(8):1689-96.

8. Gao J, Fei J, Jiang L, Yao W, Lin B, Guo H. Assessment of the reproducibility and validity of a simple food-frequency questionnaire used in dietary patterns studies. Acta Nutrimenta Sinica. 2011;33:452-6.

9. Einarsson G, Thorleifsson G, Steinthorsdottir V, Zink F, Helgason H, Olafsdottir T, et al. Sequence variants associated with BMI affect disease risk through BMI itself. Nature communications. 2024;15(1):9335.
